# Anthocyanins Recovered from Agri-Food By-Products Using Innovative Processes: Trends, Challenges, and Perspectives for Their Application in Food Systems

**DOI:** 10.3390/molecules26092632

**Published:** 2021-04-30

**Authors:** Henrique Silvano Arruda, Eric Keven Silva, Nayara Macêdo Peixoto Araujo, Gustavo Araujo Pereira, Glaucia Maria Pastore, Mario Roberto Marostica Junior

**Affiliations:** 1 Department of Food and Nutrition, School of Food Engineering, University of Campinas, Monteiro Lobato Street 80, Campinas 13083-862, Brazil; mmarosti@unicamp.br; 2 Department of Food Science, School of Food Engineering, University of Campinas, Monteiro Lobato Street 80, Campinas 13083-862, Brazil; nayarapeixoto14@hotmail.com (N.M.P.A.); glaupast@unicamp.br (G.M.P.); 3 Department of Food Engineering, School of Food Engineering, University of Campinas, Monteiro Lobato Street 80, Campinas 13083-862, Brazil; engerickeven@gmail.com; 4 School of Food Engineering, Institute of Technology, Federal University of Pará, Augusto Corrêa Street S/N, Belém 66075-110, Brazil; gapereira@ufpa.br

**Keywords:** phenolic compounds, bioactive compounds, waste valorization, emerging technologies, green chemistry, non-thermal processes, pulsed electric field, microwave, ultrasound

## Abstract

Anthocyanins are naturally occurring phytochemicals that have attracted growing interest from consumers and the food industry due to their multiple biological properties and technological applications. Nevertheless, conventional extraction techniques based on thermal technologies can compromise both the recovery and stability of anthocyanins, reducing their global yield and/or limiting their application in food systems. The current review provides an overview of the main innovative processes (e.g., pulsed electric field, microwave, and ultrasound) used to recover anthocyanins from agri-food waste/by-products and the mechanisms involved in anthocyanin extraction and their impacts on the stability of these compounds. Moreover, trends and perspectives of anthocyanins’ applications in food systems, such as antioxidants, natural colorants, preservatives, and active and smart packaging components, are addressed. Challenges behind anthocyanin implementation in food systems are displayed and potential solutions to overcome these drawbacks are proposed.

## 1. Introduction

Sustainable development plays a crucial role in improving the social, technical, and economic fields, aiming to create a circular economy and meet society’s needs. Therefore, the implementation of sustainable food production systems is essential to ensure the future needs of safe, healthy, and nutritious foods for the growing human population. In this way, the proper use of natural resources, zero food wastes, and the adoption of food production systems with high efficiency and low energy consumption are critical parts of sustainable food production [1].

Food and agricultural industries generate millions of tons of wastes and by-products annually, resulting in a significant financial burden to the processors and causing and/or contributing to immense environmental problems [2]. However, wastes and by-products derived from food processing are rich sources of potentially valuable bioactive compounds. Their sustainable use to produce value-added products/ingredients could reduce environmental issues, improve economic growth, and promote human health benefits through foods enriched with bioactive compounds [3,4]. Thereby, agri-food by-products (e.g., wastewaters, peel and seeds of fruits and vegetables, pomaces, straws, shells, brans, leaves, and other derivative by-products) have emerged as potential sources for obtaining bioactive compounds (e.g., phenolic compounds, anthocyanins, carotenoids, fiber, bioactive peptides, fatty acids, etc.) and enzymes of great interest for the food, pharmaceutical, and cosmetical industries [5,6].

Among the various value-added molecules obtained from agri-food by-products, anthocyanins have attracted special attention from people, researchers, and industries due to their multifaceted technological and biological properties. Anthocyanins are phytopigments and bioactive compounds belonging to the flavonoid class of phenolic compounds found in plant tissues (especially flowers and fruits). These water-soluble pigments provide a broad color spectrum ranging from red to blue, depending on pH [7]. This feature has been exploited to develop natural colorants [8,9] and active/smart packaging for foods [10,11]. Moreover, anthocyanins are natural antioxidants and antimicrobials that have been used as food preservatives [12,13]. In addition to their technological applications, anthocyanins have been shown to possess protective and promoting effects on human health by preventing and mitigating the onset and/or progression of neurodegenerative diseases, metabolic syndromes, cancers, etc., due to their multiple biological properties [14,15,16].

Despite the high technological, functional, and economic potential, anthocyanins’ application as food additives in food systems is still limited. Prior steps of obtaining these molecules condition it (e.g., extraction, purification, and stabilization) [17]. Anthocyanins are highly unstable compounds and easily susceptible to several factors, such as temperature, oxygen, enzymes, light (UV-visible), pH, ascorbic acid, and other substances (e.g., copigments and metals) [17,18]. Consequently, anthocyanins can be degraded by one or more of the factors mentioned above during their extraction process. Therefore, the choice of extraction technology and extraction conditions are key steps in the recovery and purification of anthocyanins for food purposes. These choices should be based on simplicity, versatility, cost, and ability to extract and preserve the target compounds [19].

The food industry is increasingly interested in innovative mild/non-thermal processing technologies (e.g., ultrasound (US), pressurized liquid extraction (PLE), supercritical fluid extraction (SFE), high hydrostatic pressure extraction (HHPE), pulsed light (PL), pulsed electric field (PEF), high voltage energy discharge (HVED), microwave (MW), enzyme-assisted extraction (EAE), and instant controlled pressure drop-assisted extraction (DIC)) for anthocyanin extraction due to their ability to enhance the extraction yield, increase the extraction rate, operate at room or low temperature, minimize the loss for thermal degradation during processing, reduce the detrimental effect on the extracted compounds, improve energy efficiency, and prioritize the use of “recognized as safe” organic solvents as compared to conventional thermal processing [2,20]. Therefore, the combination of agri-food by-products with innovative extraction techniques for obtaining anthocyanins can contribute to the achievement of high-quality products and the sustainable development of food systems.

The current review provides an overview of the main innovative processes (e.g., pulsed electric field (PEF), microwave (MW), and ultrasound (US)) used to recover anthocyanins from agri-food waste/by-products and the mechanisms involved in anthocyanin extraction, as well as their impacts on the stability of these compounds during the extraction process. Moreover, human clinical trials showing the potential beneficial health effects of anthocyanins, trends, and perspectives of anthocyanins’ applications in food systems and the challenges behind their implementation are also addressed. This review can be handy for scientists and food manufacturers from the point of view of commercial exploitation. It compiles and shows the main agri-food by-products sources of anthocyanins and the best processing conditions for their recovery using different innovative extraction technologies.

## 2. Anthocyanins

### 2.1. Chemical Structure and Properties

Anthocyanins (derived from Greek *anthos* = flower and *kyáneos* = blue) are natural plant pigments widely found in foods and beverages. These compounds are responsible for the red-orange to blue-violet colors in many fruits, vegetables, flowers, and leaves, and they are found in high quantity in the pigmented leaves, followed by fruits and flowers [21,22].

**Figure 1:**
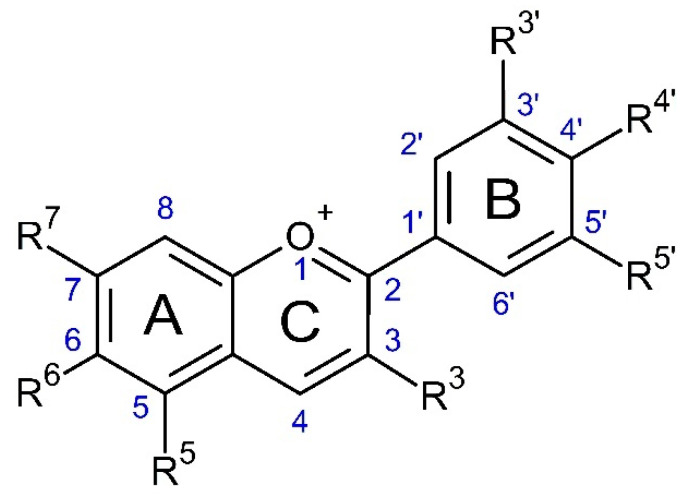
Basic anthocyanin structure.

Anthocyanins are water-soluble antioxidants pigments that belong to a polyphenol-based flavonoids family due to their characteristic C15 skeleton based on C6-C3-C6 core structure [17]. Chemically, anthocyanins are polyhydroxylated and/or polymethoxylated glycosides derived from the 2-phenylbenzopyrilium ion (flavylium cation). As can be seen in Figure 1, the basic anthocyanin structure consists of an aromatic ring (A-ring) bonded to an oxygenated heterocyclic ring (C-ring) which, in turn, is also linked through its C3 to the C1’ of a second aromatic ring (B-ring). The flavylium cation gives anthocyanins the red color that distinguishes them from other types of flavonoids [23,24].

The anthocyanin molecule is formed of an aglycone (anthocyanidin) with the presence or not of sugars (glycosylated anthocyanins) and organic acids groups (acylated anthocyanins). They can be divided into different groups based on the types of aglycones; those most commonly found in plants are cyanidin (reddish-purple color, 50%), delphinidin (red-blue color, 12%), pelargonidin (red-orange color, 12%), peonidin (magenta color, 12%), malvidin (purple color, 7%), and petunidin (red color, 7%) [25,26].

There is a wide variety of anthocyanins found in nature. Estimates point to the existence of more than 500 anthocyanins and 23 anthocyanidins [24]. The main differences between them are due to their chemical structure features, such as the number of hydroxyl groups and/or methoxyl groups present; the nature, number, and position of sugars groups linked to the anthocyanin backbone; and the presence of aliphatic or aromatic acids bonded to the sugar molecule [27,28]. The intensity and hue of the color of anthocyanins depend on the number of hydroxyl and methoxyl groups. In general, blueness increases with the degree of hydroxylation, while redness rises with the degree of methylation. Anthocyanins’ color is also influenced by copigmentation; for example, copigmentation of anthocyanidins with flavonoids improves their color intensity [26].

Anthocyanins have a positive charge on their C-ring (ionic nature) that favors the appearance of different colors in response to pH variations [29]. Polymerization, cleavage, and derivatization are mainly reactions that occur with anthocyanins according to pH changes and are related to their color stability, resulting in browning compounds, colorless, and colored molecules, respectively [18]. In general, highly acidic conditions favor redness color and the stability of the anthocyanins due to a higher formation of flavylium cations. In contrast, alkaline conditions reduce stability, increase blueness color, and promote the chemical degradation of the anthocyanins [25].

**Figure 2:**
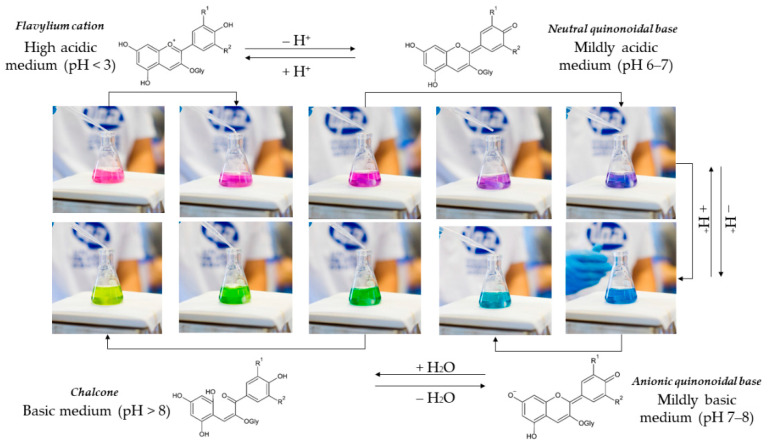
pH-dependent chemical structures and color changes of anthocyanins from red cabbage. Experiment performed by Gustavo Araujo Pereira and registered by Karla Ferreira Nery Martins (photos). HCl and NaOH (0.1 M) were employed to alter the pH of the red cabbage aqueous extract.

In superacid conditions (pH < 3), the flavylium cation is the majority species and favors reddish-purple color. At increasing pH conditions (pH 4–5), colorless carbinol pseudo base predominates, and anthocyanin solution become very little colored due to the small number of other colored species (flavylium cation and quinonoidal anion). At mildly acidic conditions (pH 6–7), the blue-violet neutral quinonoidal species are predominant. As the pH increases (pH 7–8), the neutral quinonoidal species’ deprotonation forms anionic quinonoidal bases that promote an intense blue color. In superalkaline conditions (pH > 8), yellowish chalcone is formed and, subsequently, anthocyanins undergo breakdown according to their substituent groups and structure (see Figure 2) [25,26]. Figure 2 shows the pH-dependent structural and color changes of anthocyanins from a red cabbage aqueous extract.

### 2.2. Natural Sources

Anthocyanins are naturally found in a wide number of foods, including fruits and vegetables. They are predominantly found in berries, currants, grapes, tropical fruits, red to purplish blue-colored leafy vegetables, grains, roots, and tubers. The berries are the wealthiest and most well-known sources of anthocyanins, such as blackberries, blueberries, strawberries, blackcurrants, cherries, chokeberries, elderberries, and gooseberries. Furthermore, there are other anthocyanin sources, such as red grapes, eggplant, acai, jabuticaba, oranges, purple corn, red wine, red cabbage, apples, radishes, pomegranate, black carrots, purple potatoes, and edible flowers (e.g., red hibiscus, red rose, red pineapple sage, red clover, pink blossom, cornflower, blue chicory, blue rosemary, purple mint, purple passionflower, purple sage, common violet, and lavender) [25,26]. In addition to being widely consumed in fresh and frozen forms, anthocyanins-rich fruits/vegetables are also found in several processed and derived products, including wine, dried and canned fruits, yogurts, beverages, jams, and jellies [30]. Anthocyanin profile and content varies between the foods due to external and internal factors, such as biotic and abiotic stress, cultivation practices, genetics, intensity and type of radiation, temperature, processing, and storage [30,31,32].

Various phenolic compounds remain trapped in the food matrix during food processing, resulting in high value-added agri-food by-products discarded as natural waste in the environment or used for composting. This generates great economic losses and environmental problems that could be avoided using an agri-food by-product recovery system. In general, the agri-food by-products are mainly constituted of skin/peel and seeds, and they are generated mainly from fruits and vegetable processing [33]. The main and best-known agri-food by-product is the grape pomace that results from grape juice and winemaking processing, consisting mainly of skin, seeds, stems, and remaining pulp. Grape pomace stores many phenolic compounds. It is the most-used raw material for anthocyanin extraction by the food industry [33,34].

Although anthocyanin content varies according to the cultivation techniques and species, the malvidin was the major anthocyanin (0.29 to 3.33 mg/g skin) in all studied grape cultivars, followed by petunidin, delphinidin, peonidin, and cyanidin [35]. Thirteen anthocyanins were identified from grape pomace by HPLC-MS/MS, whose results also confirmed that malvidin is the predominant anthocyanin. The most intense chromatographic signals observed were for malvidin-3-*O*-glucoside, malvidin-3-*O*-acetylglucoside, and malvidin-3-*O*-*p*-coumaroylglucoside [36]. Meanwhile, grape seeds showed only two anthocyanins that were found in low concentrations, namely malvidin-rutinoside (0.024 µg/g extract) and malvidin-hexoside (0.010 µg/g extract) [37]. Another anthocyanin source resulting from the winemaking industry is the wine lees that, according to some authors, could be alternative that is cheaper and more easily extracted than the traditional sources [38].

Other agri-food by-products have been exploited for anthocyanin extraction, such as black carrot pomace [39], eggplant by-product [40,41], black soybean seed coat [42], purple corn cob and corn husk [43], onion peel [44], black rice bran [45], and some berry by-products (e.g., blueberry [46], red raspberry [47], sweet cherry [48], sour cherry [49], bilberry [50], blackcurrant [51], blackberry [52], jabuticaba [53], black chokeberry [54], and mulberry [55], among others). Nonetheless, they are less explored and less known when compared to grape pomace. Therefore, research should be intensified and driven towards the less exploited agri-food by-products to obtain high added-value anthocyanin extracts.

### 2.3. Chemical Stability

Anthocyanins are highly unstable and susceptible to degradation, which varies widely according to their chemical structure and food concentration. This strongly influences the anthocyanin content in the final product and their benefits to human health [56]. Several environmental factors affect anthocyanin color and stability. The most important and studied of them are temperature, pH (see Section 2.1), enzymes, light, oxygen, metallic ions, sulfites, and interaction with other food components (e.g., other flavonoids and phenolics, ascorbic acid, sugars, etc.) as well as other factors [18,32].

Anthocyanins are largely unstable during food processing, in which the temperature can reach around 50–150 °C [57]. Thermal processes are one of the main and most well-known factors that influence the anthocyanin stability rate. They are extremely sensitive to thermal treatment. When exposed to high temperatures and prolonged heating, the anthocyanins undergo oxidation and structure breakdown characterized by successive reactions of deglycosylation, nucleophilic attack of water, cleavage, and polymerization. These structural changes result in the loss of sugar moieties, color, stability and, consequently, considerably reduce anthocyanin content in the final product [32]. Anthocyanin stability is maintained at temperatures up to 60 °C. High processing temperatures (>60 °C) promote the anthocyanin molecules’ breakdown into colorless compounds. Therefore, prolonged exposure to high temperatures should be avoided to reduce the chalcone formation and losses of anthocyanin stability [58]. The lower the processing temperature, the greater the accumulation and stability of anthocyanins (flavylium cation).

Anthocyanins are enzymatically degraded in foods due to glycosidases and polyphenol oxidases that promote their decolorization. The β-glycosidases hydrolyze glycosidic bonds between the glycosyl residue and an anthocyanins aglycone, releasing their less stable counterpart, anthocyanidins. On the other hand, in the presence of oxygen, polyphenol oxidases catalyze the hydroxylation of monophenols to *o*-diphenols and the oxidation of *o*-diphenols to *o*-quinones compounds promoting anthocyanin degradation and catalyzing the formation of brown-colored compounds. Furthermore, peroxidases have also been reported to promote anthocyanin degradation [59,60]. Therefore, the inactivation of these enzymes can be a good strategy to improve anthocyanin stability.

The accumulation of anthocyanins is also directly influenced by their exposure to light. In plants, this exposure favors their biosynthesis, resulting in colored pigments, while over food processing/storage, light accelerates their degradation. Light intensity and spectrum influence the anthocyanin biosynthetic genes, improving their production. In contrast, light irradiation promotes anthocyanin molecule excitation, leading to the formation of carbon-centered radicals that can generate peroxyl radicals in the presence of oxygen, accelerating anthocyanin degradation [61,62].

Oxygen is another major environmental factor influencing anthocyanin stability, and that can result in some undesired outcomes. Anthocyanins undergo decomposition under aerobic conditions. The molecular oxygen promotes harmful effects in the anthocyanin molecule [60]. In the presence of oxygen, ascorbic acid-induced degradation of anthocyanin results in the formation of hydrogen peroxide and oxidative cleavage of the C-ring, producing brown pigments that are undesirable changes in food, especially in juices [63]. Moreover, the synergism between oxygen and high temperatures is the factor that most accelerates anthocyanin degradation [64]. The removal of oxygen protects the anthocyanins against oxidative degradation and slows down thermal degradation.

The interaction between anthocyanins and sulfites leads to the formation of colorless adducts because of the interruption of the conjugated π-electron system [18]. As a consequence, sulfite addition should be avoided in anthocyanin-rich processed products.

On the other hand, anthocyanin association reactions can improve their stability and color by protecting C2 of the flavylium chromophore from the nucleophilic attack of water. These associations can occur in three ways: (1) self-association (anthocyanins are associated with each other via hydrophobic interactions that occur between their aromatic nuclei), (2) copigmentation (anthocyanins are associated with other compounds, such as other phenolic compounds), and (3) metal complexing (anthocyanins are associated with metals, such as magnesium and aluminum, via interactions of their *o*-hydroxy groups). Meanwhile, copigmentation can be intramolecular or intermolecular. Intramolecular copigmentation is characterized by stacking the hydrophobic acyl moiety covalently bound to sugar and the flavylium nucleus. In contrast, intermolecular copigmentation occurs when anthocyanins interact with some colorless compounds (e.g., aurones, flavones, and flavanols) through van der Waals interactions between the planar polarizable nuclei of the anthocyanin with these compounds [17,32]. Therefore, the copigments approach can be a promising tool for improving anthocyanins color and stability.

As a general remark, food processing should minimize these aforementioned undesirable changes to obtain high-quality anthocyanins-rich foods/extracts.

### 2.4. Biological Activities

Anthocyanins are widely studied as bioactive agents to manage and/or prevent the onset/development of several diseases, such as chronic degenerative diseases including cardiovascular diseases, cancers, type 2 diabetes mellitus, neurodegenerative diseases, and dyslipidemias [28]. Several biochemical parameters have been related to the prevention or development of these diseases. Some biochemical parameters, such as tumor necrosis factor-alpha (TNF-α), interleukins (ILs), nuclear factor-kappa B (NF-kB), and cyclooxygenase 2 (COX-2), are involved in the inflammatory responses. In contrast, the increase in reactive oxygen species (ROS) and malonaldehyde, and the reduction in the activity/expression of antioxidant enzymes, such as superoxide dismutase (SOD), catalase (CAT), etc., are related to oxidative processes [65,66,67].

The biological activities of anthocyanins are dependent mainly on their structure and chromatic features. For example, the three hydroxyl groups in the B-ring in the molecular structure of the delphinidin increase their efficiency against cancer cells compared with other anthocyanins, such as cyanidin [28].

Beneficial effects for human health related to anthocyanins have been demonstrated in human clinical trials. Daily intake of anthocyanins improved glycemia, insulin sensitivity, lipids and lipoproteins profiles, memory function, cardiovascular health, antioxidant status, gut microbiota composition, etc. (for more details, see Table 1).

After daily intake of 320 mg of purified anthocyanins for 12 weeks, diabetics and prediabetic subjects showed improvement in insulin secretion, insulin sensitivity, and lipid profile. The results showed a reduction in glycated hemoglobin A1c level (HbA1c), low-density lipoprotein cholesterol (LDL-cholesterol), and apolipoprotein B (ApoB), while increasing ApoA1. These effects may be attributed to the molecular mechanisms of glycolipid metabolism, such as activation of adenosine monophosphate-activated protein kinase (AMPK) that inhibit 3-hydroxy-3-methylglutaryl-coenzyme A (HMG-CoA) reductase (limiting enzyme of cholesterol synthesis), or by regulating transcriptional factor Forkhead box O1 (FoxO1) of adipose triglyceride lipase (main lipase involved in triglycerides breakdown in adipocytes), inhibiting cholesterol synthesis and adipocyte lipolysis [68,69].

Human clinical trials further support that purified anthocyanins may improve adipocyte dysfunction by regulating adipokines expression (e.g., adipsin and visfatin). Adipsin plays an important role in maintaining the pancreatic β-cell function, whose failure in the human system promotes the deficiency in adipsin and, therefore, results in insulin resistance and the progression of type 2 diabetes mellitus. Meanwhile, increased visfatin expression is associated with obesity and type 2 diabetes mellitus. The daily administration of 320 mg purified anthocyanins for 12 weeks increased the serum adipsin and reduced the serum visfatin in diabetic patients. Besides that, it significantly improved the HbA1c and ApoA1 and decreased C-peptide, C-peptide index, and ApoB [69].

Previous human clinical studies have suggested that patients with metabolic syndrome showed significatively reduced inflammation and improved lipid profile, evidenced by decreased serum cholesterol, LDL-cholesterol, triglycerides, fasting blood glucose, and inflammatory biomarkers after daily intake of 320 mg of purified anthocyanins for 4 weeks. These beneficial effects have been associated with anthocyanins’ capacity in regulating the expression/activation/activity of pro-inflammatory mediators, such as NF-κB signaling pathways, pro-inflammatory cytokines (e.g., TNF-α, IL-6, and IL-1A), and pro-inflammatory enzymes (e.g., COX-2). The anthocyanins also inhibited the production of pro-inflammatory molecules, such as high-sensitivity C-reactive protein (hs-CRP), the best validated inflammatory biomarker, while increasing SOD expression. Moreover, anthocyanin supplementation upregulated proliferator-activated receptor-γ (PPAR-γ) expression. PPAR-γ plays a crucial role in lipid and glucose homeostasis by modulating dietary fats and glucose metabolism, adipocyte differentiation, and inflammatory responses [77,78].

Dietary supplementation studies have shown that intake of increasing anthocyanin concentrations for 12 weeks significantly improved the antioxidant status in patients with dyslipidemia through the reduction in oxidative stress-related biomarkers, such as malonaldehyde, urine 8-iso-prostaglandin F_2α_ (8-iso-PGF_2α_), and urine 8-hydroxy-2′-deoxyguanosine (8-OHdG) and promoted the increase in the total SOD activity. Furthermore, anthocyanin supplementation also improved the anti-inflammatory capacity through decreased inflammatory cytokines expression (e.g., IL-6 and TNF-α), improved lipids profile, and cholesterol efflux capacity due to the reduction in ceramide species levels [65,73].

The consumption of anthocyanins-rich cherry juice (about 138 mg of anthocyanins/day) for 12 weeks has been shown to attenuate cognition losses in older adults with mild-to-moderate dementia Alzheimer’s type by improving cognitive tasks, such as verbal fluency and short and long-term memory [88]. Daily intake of purified anthocyanins (320 mg/day for 16 weeks), anthocyanins-rich black rice extract (19.08 mg of anthocyanins/day for 12 weeks), and blueberry anthocyanins (258 mg of anthocyanins/day for 16 weeks) also improved the cognitive performance in subjects with subjective memory impairment or mild cognitive impairment [81,86,87]. Anthocyanins can eliminate and block the action of free radicals in the brain, protect neurons susceptible to inflammatory processes, enhance existing neuronal function, increase cerebrovascular blood flow, and stimulate neurogenesis in areas of the brain related to cognition, among other mechanisms [15,88]. However, there is no evidence that anthocyanins can halt disease progression [88].

Clinical trials have also demonstrated that anthocyanin supplementation can improve cardiovascular function [79,80,84,89,91], modulate gut microbiota composition [90,91], alleviate ulcerative colitis symptoms [67], enhance exercise recovery effectiveness [66], reduce ocular fatigue [92], and maintain a healthy skin facial condition [93]. Therefore, the consumption of anthocyanins-rich fruits/extracts showed beneficial effects on glucose and lipid metabolism, oxidative stress, inflammatory cascade, and gut microbiota profile and, thereby their daily intake may have a key role in the prevention or treatment of type 2 diabetes mellitus, obesity, dyslipidemias, metabolic syndrome, neurodegenerative disorders, cardiovascular diseases, cancers, and other chronic degenerative diseases, as well as in maintaining overall health and wellbeing. Nevertheless, further research is required to elucidate the effective anthocyanin concentrations required to perform their biological effects and action mechanisms of anthocyanins in the body.

## 3. Innovative Processes for Anthocyanin Extraction from Agri-Food By-Products

### 3.1. Pulsed Electric Field

Pulsed electric field (PEF) is an innovative non-thermal processing technology regarded as environmentally friendly due to its low energy expenditure water depletion [1]. PEF technology has been successfully used to extract anthocyanins from different matrices [94,95,96,97,98]. As shown in Table 2, PEF technology improved anthocyanin extraction from different agri-food by-products compared to conventional extraction technologies or even other innovative extraction technologies (e.g., US and high voltage electrical discharges). The potentiation of anthocyanin extraction promoted by PEF technology is due to the increase in mass transfer and, consequently, anthocyanins release into the medium, caused by the formation of temporary (reversible) or permanent (irreversible) pores in the cell membranes, a phenomenon known as electroporation [17].

Cell membranes are the cell barriers that govern the target compounds’ extraction yield. Thus, extraction rate depends on the cell membrane permeability [107]. The cell membrane acts like a capacitor with a low dielectric constant, in which the cell membrane conductivity is extremely lower than that of the surrounding medium and cytoplasm. However, when the cell is exposed to a strong external electric field, ions migrate from the fluid and accumulate at the cell membrane interface, forming free charges of opposite sign at the two interfaces (inner/outer) of the membrane itself that increases transmembrane potential on the cell surface. Due to the very low thickness of a typical plant cell membrane (approximately 5 nm), electrostatic attraction of opposite charges occurs along the cell membrane, inducing cell membrane compression, reducing the membrane thickness. When the electric field strength exceeds a certain critical limit (E_c_), usually around 0.2–1 V/m for plant cells, the elastic properties of the cell membrane does not resist the electrostatic attraction that leads to reversible (E ≈ E_c_) or irreversible (E >> E_c_) formation of micropores in weaker areas of the cell membrane, increasing cell permeability. Thereby, PEF treatment enhances the migration of target compounds located in the cytoplasm across the cell membrane, which boosts mass transfer and, consequently, increases the extraction rates and yield [108,109].

Plant cell membrane permeabilization is easier to reach than in microbial cells due to their larger cell size, requiring lower critical electric field strengths (0.5–2 kV/cm) for electroporation and, consequently, lower energy consumption [1,108]. The PEF extraction process is based on the direct application of very short duration pulses (usually micro to milliseconds) of current high-electric voltage to a matrix placed between two electrodes [2]. Electric field strength, treatment time, specific energy input, pulse number, and temperature are the main parameters that affect the performance of PEF extraction. Overall, electroporation intensifies as the intensity of these parameters increases and, thereby enhancing the target compounds extraction [107,110]. Indeed, some studies have shown an increase in anthocyanin extraction from agri-food by-products as these parameters are intensified. When evaluating the PEF treatment effect on blueberry by-product, Pataro et al. [46] reported that anthocyanin extraction enhances (up to 75%) as specific energy input increases (1–10 kJ/kg). In this study, PEF technology did not affect the number and type of anthocyanins extracted and did not induce any degradation/modification of individual anthocyanins [46]. Similar findings were obtained by Bobinaitė et al. [102]. They demonstrated that the higher the electric field intensity (1–5 kV/cm) applied, the greater the anthocyanin extraction from the blueberry by-products (up to 95% higher than conventional extraction). In particular, malvidin and peonidin glycosides extraction were not significantly affected by electric field intensity, while delphinidin, cyanidin, and petunidin glycosides significantly increased when higher electric field intensities were applied. Moreover, it is worth pointing out that the authors did not report PEF technology’s effect on the number, type, and stability of individual anthocyanins extracted [102]. In another study, Lončarić et al. [103] verified that the intensification of electric field intensity (10–20 kV/cm) and pulse number (10–100) enhances anthocyanin recovery from blueberry pomace. Moreover, PEF technology was more efficient at extracting anthocyanins from blueberry pomace than other innovative technologies (US and high voltage electrical discharges) [103]. Likewise, Zhou et al. [100] reported enhanced anthocyanin recovery from blueberry by-product by PEF technology compared to US extraction. In this study, anthocyanin extraction improved as pulse number (up to 10 pulses) and electric field intensity (up to 20 kV/cm) increased [100]. Similarly, PEF technology was more effective for anthocyanin extraction from grape pomace than conventional (up to 430%), US (up to 22%), and high voltage electrical discharges (up to 55%) extraction. This study evidenced that the higher the specific energy input applied, the greater the total and individual anthocyanins (delphinidin-3-glucoside, petunidin-3-glucoside, peonidin-3-glucoside, and malvidin-3-glucoside) extraction [105]. These findings agreed with Medina-Meza and Barbosa-Cánovas [106] and Corrales et al. [95]. They indicated higher anthocyanin yields from grape pomace using PEF treatment than control, US, and high hydrostatic pressure extractions. Moreover, PEF treatment of higher intensity (289.8 W and 25.2 pulses) resulted in better extractability of anthocyanins from grape pomace [106].

Nonetheless, the PEF extraction process intensification does not always improve anthocyanin extraction yields. Lamanauskas et al. [47] observed that the PEF treatment of red raspberry by-products increased anthocyanin extraction up to 25.7% compared to conventional extraction. However, the intensification of electric field intensity (1–3 kV/cm) and specific energy input (1–12 kJ/kg) did not promote any significant modification in the content of anthocyanins extracted. PEF treatment of sweet cherry by-product increased anthocyanin extraction by up to 38.4%, with no effect on the number and type of anthocyanins extracted. Moreover, the anthocyanin yield remained unchanged as the electric field intensity increased (0.5–3 kV/cm) [48]. PEF-treated sour cherry by-product had an anthocyanin yield 44–54% higher than conventional extraction and electric field intensity (1–5 kV/cm) did not significantly influence anthocyanin extraction [99]. Likewise, PEF treatment improved total and individual anthocyanins (delphinidin-3-glucoside, petunidin-3-glucoside, peonidin-3-glucoside, and malvidin-3-glucoside) extraction from grape pomace by up to 18.9%, but electric field strength intensification (1.2–3 kV/cm) did not allow for any significantly higher anthocyanin extraction yield [104].

Depending on the PEF extraction process intensity, there may be a reduction in anthocyanin extraction yields with the PEF process intensification. Increasing the intensity of some PEF extraction process parameters (e.g., electric field intensity and specific energy input) showed a tendency to reduce anthocyanin recovery from red raspberry by-product [47] and sweet cherry by-product [48]. When evaluating the PEF treatment effect on blueberry by-product, Zhou et al. [100] reported that anthocyanin extraction enhances up to a certain point as pulse number (up to 10 pulses) and electric field intensity increases (up to 20 kV/cm). However, the application of higher electric field intensity (>20 kV/cm) and pulse number (>10 pulses) drastically and progressively reduced anthocyanin extraction [100]. PEF application increased anthocyanin extraction from peach pomace by up to 11.8-fold compared to conventional thermal extraction. However, intensification of specific energy input (0.02–20 kJ/kg) drastically and progressively reduced anthocyanin extraction yields in PEF technology. These findings suggest that anthocyanins from peach pomace are highly sensitive to degradation by PEF treatment intensification with a degradation constant equal to 8.2 kg/kJ [98]. In another study, Pataro et al. [48] noticed that the cyanidin-3-glucoside content extracted from sweet cherry by-product decreases as the electric field intensity increases (0.5–3 kV/cm).

In general, the intensification of PEF extraction process parameters promoted higher anthocyanin extraction yields from blueberry and grape by-products. At the same time, it did not affect anthocyanin recovery from red raspberry, sweet cherry, and sour cherry by-products. On the other hand, PEF intensification had negative effects on anthocyanin extraction from peach pomace. Overall, electric field intensity and specific energy input applied to the agri-food by-products ranged from 0.5–20 kV/cm and 0.02–20 kJ/kg, respectively. Nevertheless, the behavior of anthocyanin extraction greatly differed according to the matrix as mentioned above. The influence of PEF treatment on anthocyanin extraction depends on distinct factors, such as the relative location in the plant cell, cell size distribution between peel and pulp, ability to bind to the matrix, chemical structure, stability, etc. [46,99]. Peel cells are lower than pulp cells. Thus, it is likely that a lower degree of cell disintegration occurs in peel cells, making anthocyanin extraction from peels more difficult [46]. Monoglucosides anthocyanins are more easily extracted than acylated glucoside anthocyanins since the latter seems to be physically entrapped within the matrix or form hydrogen bonds with cell wall polysaccharides [95]. When evaluating the effect of PEF treatment (10 kV/cm electric field intensity, 5 μs pulse width, and 10 Hz pulse frequency) on individual anthocyanins, Sun et al. [111] verified that cyanidin-3-glucoside was more susceptible to PEF degradation than cyanidin-3-sophoroside. In addition to sugar moieties and phenolic groups bonded to the C-ring, anthocyanin stability depends on -OH and -OCH_3_ groups at position R1 and C3′ and R2 and C5′ from the B-ring. The anthocyanin stability enhances as the number of -OH, -OCH_3_ and acyl groups increases (stability order: malvidin > peonidin > petunidin> cyanidin > delphinidin) [95]. Electrode materials from PEF devices have also been shown to influence anthocyanin recovery. Sun et al. [111] noticed that stainless steel was the electrode material that allowed greater anthocyanin retention after PEF treatment. In contrast, pure titanium and titanium-based alloy materials led to greater anthocyanin degradation. Moreover, PEF technology can show a very distinct effect in different fruit varieties of the same species [112]. Furthermore, the critical electric field strength required to promote electroporation in cell membranes can differ for each agri-food by-product. As discussed previously, the closer the electric field strength values to the critical value (E_c_), the greater the extent of the electroporation phenomenon and, consequently, higher compounds release into the extraction medium [108,109]. However, when the electric field strength values overcome the critical value (E_c_), the excess energy can promote structural changes and/or degradation of organic molecules [100]. Thereby, the use of excessively high electric field strengths can lead to drastic and progressive anthocyanin degradation.

The results compiled here demonstrate that high electric field strengths are demanded to promote complete electroporation of cell membranes from blueberry and grape by-products. In contrast, moderate electric field strengths are required for red raspberry, sweet cherry, and sour cherry by-products, and low electric field strengths for peach pomace. Anthocyanins are mainly located in peels of blueberry and grape, and there is a great difference in peel and pulp cell size. Therefore, anthocyanins from smaller and denser cells of peels are released more difficulty than those from larger and juicer cells of pulp [46]. As blueberry and grape by-products are mainly composed of peels, higher electric field strengths are required to promote peel cell disintegration and consequent anthocyanin release. Zhou et al. [100] showed that very high electric field strengths are needed to cause anthocyanin degradation from blueberry by-products. In contrast, increasing extraction yields were reported by Barba et al. [105] for grape pomace anthocyanins even when a high electric field intensity (13.3 kV/cm) was applied together with increasing specific energies input (0–564 kJ/kg). Meanwhile, moderate PEF treatment can affect anthocyanin stability from sweet cherry by-product during extraction since cyanidin-3-glucoside content progressively reduced as the electric field intensity increased (0.5–3 kV/cm) [48]. On the other hand, Plazzotta et al. [98] proved that low electric field strengths (<0.8 kV/cm) are needed to extract anthocyanins from peach pomace since the application of electric field strengths higher than 0.8 kV/cm drastically degraded its anthocyanins. Studying the anthocyanin degradation kinetics, the authors found a high degradation constant (8.2 kg/kJ), showing that peach pomace anthocyanins are very sensitive to PEF treatment intensification [98]. Similarly, previous studies demonstrated that degradation constants of cyanidin-3-glucoside exposed to PEF treatment were boosted as electric field strength increased [113,114]. When the electric field strength is high enough, electrochemical reactions can occur (particularly electrolysis of solvent and electrode corrosion), leading to the production of high concentrations of metallic ions and reactive oxygen species (especially hydroxyl radicals and hydrogen peroxide). Metallic ions, it turns, can catalyze the decomposition of hydrogen peroxide into hydroxyl radicals via the Fenton reaction. Hydroxyl radicals have been identified as the main reactive oxygen species related to benzene ring cleavage in phenolic compounds [107,111]. Indeed, increased hydroxyl radicals formation has been observed after PEF treatment [111,115]. Sun et al. [115] proposed a mechanism for the PEF-induced cyanidin-3-sophoroside degradation based on the action of reactive oxygen species formed by electrochemical reactions at the electrode-medium interface during PEF treatment. According to the authors, hydrogen peroxide promotes a nucleophilic attack at the C2 of the C-ring leading to the cleavage of the heterocyclic ring between C2 and C3 through the Baeyer–Villiger oxidation reaction and consequent formation of benzoyloxyphenylacetic acid ester of cyanin type (called cyanone). Then, cyanone is oxidized by other oxidants (e.g., hydroxyl radicals) at either of two phenolic hydroxyl groups on A-ring forming several quinonoid cyanone isomers. Zhang et al. [113] verified that protocatechuic acid, 2,4,6-trihydroxybenzoic acid, and eight other unknown compounds were the main degradation products of cyanidin-3-glucoside exposed to PEF treatment. Protocatechuic acid, phloroglucinaldehyde, a dimer, chalcone-3-sophoroside, and four quinonoid cyanone isomers were identified by Sun et al. [115] as the main PEF degradation products of cyanidin-3-sophoroside. These studies also pointed out that the degradation mechanism and pathways of anthocyanins by PEF treatment were different from thermal treatment [113,115]. Therefore, the reactive oxygen species’ action on anthocyanins could explain their degradation during PEF processing, particularly when high electric field strengths are employed.

In addition to enhancing the anthocyanin recovery from agri-food by-products without affecting the number and type of anthocyanins extracted [46,48,102,103,104], PEF technology can be used to improve the extraction of individual anthocyanins selectively. For example, the PEF treatment of grape pomace remarkably enhanced the monoglucoside anthocyanin extraction compared to acylated glucoside anthocyanins [95]. Bobinaitė et al. [102] observed that higher PEF treatment intensities particularly increased the extraction of delphinidin, cyanidin, and petunidin glycosides from blueberry by-products. Barba et al. [105] found PEF treatment intensification remarkably enhanced malvidin-3-glucoside extraction compared to the content of other individual anthocyanins. Moreover, PEF technology can be more selective to extract anthocyanins rather than other phenolic compounds. Brianceau et al. [104] noticed that PEF treatment increased the total anthocyanins/total flavan-3-ols ratio regarding the non-treated sample in grape pomace. Likewise, Barba et al. [105] verified that PEF-treated grape pomace had a higher total anthocyanins/total phenolic compounds ratio than reference extraction.

Finally, the application of high-intensity PEF technology to extract anthocyanins from agri-food by-products can lead to some anthocyanin degradation. Nevertheless, low/moderate-intensity PEF treatments improved anthocyanin recovery without inducing any anthocyanin degradation/modification. Therefore, PEF is a promising innovative technology to extract anthocyanins from agri-food by-products.

### 3.2. Microwave

Microwave (MW) is another innovative and green extraction technology considered to be more advantageous than conventional extraction technologies due to its high extraction rate, less use of solvents, and shorter extraction time [109]. Anthocyanins from different plant sources have been successfully extracted by using MW technology [116,117,118,119,120]. MW technology enhanced anthocyanin extraction from various agri-food by-products compared to conventional extraction technologies or even other innovative extraction technologies (e.g., US) (for more details, see Table 3). MW technology improves anthocyanin extraction due to its ability to increase mass transfer rates and, consequently, anthocyanins release into the extraction medium because of the cell interruption caused by internal overheating [17].

As discussed earlier, cell membranes are the cell barriers that control solute permeability and, therefore, extraction yields. Thus, cell rupture is needed to boost anthocyanin extraction from plant matrices. When microwave irradiation is applied to a matrix or extraction medium, microwave energy is absorbed by polar compounds causing ion conduction and dipole rotation [109]. In ionic conduction, the electric field produced by the microwaves induces electrophoretic migration of charge carriers (e.g., ions and electrons), generating friction between the moving ions and the medium that causes heating. Meanwhile, in dipole rotation, heating occurs due to the collision between dipolar molecules and surrounding molecules caused by the dipolar species’ oscillation when they attempt to align themselves with the alternating electric field produced by microwaves [128]. In plant matrices, the water naturally present inside their structure selectively absorbs the microwave energy favoring localized heating above or near the boiling point of water. This internal overheating causes the expansion and rupture of cell structures (especially cell walls and cell membranes) by disrupting the dipole attractions, hydrogen bonds, and van der Waals forces, which facilitates the release of target compounds bound to the matrix and solvent penetration into the plant materials, resulting in the improved mass transfer of these compounds into the extraction medium [17,109]. Moreover, MW-induced temperature rise modifies the solvent properties, reducing its viscosity and surface tension while increasing its diffusivity and molecular mobility, improving the mass transfer rates [19].

MW extraction process is based on the focused or non-focused application of non-ionizing electromagnetic waves of frequency ranging from 300 MHz to 300 GHz on a sample [17]. Solvent type is a crucial parameter in MW extraction since its ability to solubilize the target compounds and absorb microwave energy is critical for recovering these compounds. Regarding the solubility, it is preferable to use solvents that have the Hildebrand solubility parameter (measure the cohesion (interaction) energy of the solvent-solute mixture) similar to those of the target compounds [128]. On the other hand, the ability of a solvent to absorb electromagnetic energy and dissipate heat depends on its dielectric constant (*ε*′) and dielectric loss factor (*ε*″). The dielectric constant is a parameter proportional to the amount of energy absorbed. Meanwhile, the dielectric loss factor denotes a solvent′s ability to dissipate input dielectric energy as heat. Polar solvents with a high dielectric constant (e.g., water and ethanol, *ε*′ = 78.5 and 24.3 at 25 °C, respectively) have a better capacity to absorb electromagnetic energy and reemit it as heat, whereas apolar solvents with a low dielectric constant (e.g., hexane, *ε*′ = 1.87 at 25 °C) are almost insensitive to this energy, besides not being considered green solvents [128,129]. Thereby, solvents with a high dielectric constant are recommended for MW extraction purposes. Nevertheless, the relationship between dielectric properties from the plant material and solvent is also essential. When plant material has better dielectric properties than solvent, that is, when the dielectric loss tangent (*δ* = *ε*′/*ε*″) of plant material is higher than the solvent, the plant material better absorbs the microwave energy than the solvent. This allows plant material to reach higher temperatures than the solvent, increasing the inside cell pressure that results in the cell membrane’s rupture and the release of target compounds into the extraction solvent [128]. This factor can be managed by changing the extraction temperature. Lee et al. [130] reported that the dielectric loss tangent of plant material (okra) raised as temperature increased, while for the solvent (water), this parameter reduced as temperature increased. Similar outcomes were found by Mao et al. [131] for orange, apple, and mango pomaces. Therefore, higher extraction temperatures can improve the dielectric properties of plant material regarding the solvent.

In addition to solvent, microwave power, irradiation time, and extraction temperature also significantly impact the performance of MW extraction [132]. Overall, cell structure disintegration intensifies as the intensity of these parameters increases, thereby improving the extraction yields of target compounds [119]. Indeed, several studies conducted with MW have shown an increase in anthocyanin extraction from agri-food by-products as these parameters are intensified. When evaluating the MW treatment effect on eggplant peel, Doulabi et al. [40] reported that the anthocyanin extraction enhances as the microwave power increases (100–300 W). Şahin et al. [49] verified that anthocyanin recovery from sour cherry peels gradually enhanced as the microwave power (350–500 W) and irradiation time (0.5–1.5 min) increased. Similarly, the intensification of microwave power (600–1000 W) and irradiation time (up to 7 min) progressively increased anthocyanin recovery from grape pomace [124]. Likewise, the higher the microwave power (300–600 W), the greater the anthocyanin extraction from bilberry pomace [50]. Grape pomace also had higher anthocyanin yields as the microwave power increased (100–300 W) [126]. Meanwhile, longer irradiation times gradually improved anthocyanin recovery from wine lees (30–90 s at 300 W) [133], sour cherry pomace (30–90 s at 900 W) [134], and saffron floral bio-residues (0.5–5 min at 800 W) [135].

Although the MW intensification generally enhances the anthocyanin extraction, a decreased anthocyanin recovery can occur depending on the intensity of the process parameters. Grillo et al. [121] reported that anthocyanin extraction from blueberry peel enhances up to a certain point (up to 62.7% higher than conventional extraction) as extraction temperature (up to 60 °C) and irradiation time (up to 15 min) increases and decreases afterward. Similar behavior was observed by Backes et al. [123] for fig peel anthocyanins, where the extraction yields enhanced (38% and 16.73% compared to the US and conventional thermal extractions, respectively) as the extraction temperature was increased (up to 64.21 °C), but begins to decrease with the rise of extraction temperature (>64.21 °C) and irradiation time (>5 min). In another study, Kumar et al. [42] showed that anthocyanin recovery from black soybean seed coat progressively raised (up to 4.72-fold compared to conventional solvent extraction) with increasing microwave power. However, it diminished at microwave powers greater than 510 W. Likewise, anthocyanin yields from blackcurrant bagasse were boosted as the microwave power (up to 551 W) and irradiation time (up to 16.4 min) increased and, then, it begins to decrease [51]. The anthocyanin extraction from grape pomace also improved as the microwave power and time extraction increased. However, when it was applied microwave powers and irradiation times higher than 428 W and 2.23 min, respectively, anthocyanin extraction gradually decreased [125]. Similar behavior was obtained by Halee et al. [45] for anthocyanins from black rice bran, in which enhanced anthocyanin yields were achieved as the microwave power (up to 648 W) and irradiation time (up to 83 s) increased and subsequently declined. Meanwhile, Ferreira et al. [122] pointed out that an increase in microwave power (from 525 to 700 W) led to lower anthocyanin recovery from blueberry bagasse. Increased microwave power (from 700 to 1000 W) also caused a reduction in anthocyanin extraction from onion peel, while longer irradiation time (5 min) improved it [44]. MW application increased anthocyanin extraction from peach pomace by up to 26-fold compared to control extraction. However, the intensification of microwave power (180–900 W) drastically and progressively reduced anthocyanin extraction yields, while irradiation time (10–50 s) had the opposite effect [127]. Higher anthocyanin yield was also found in MW-treated black carrot pomace compared to conventional (133%) and US (24.12%) extractions. Nevertheless, it was noted that increasing microwave powers (340–680 W) and irradiation times superior to 9.86 min harmed anthocyanin recovery [39]. Doulabi et al. [40] and Backes et al. [136] noticed that the longer the irradiation time, the lower the anthocyanin recovery from eggplant peel and fig peel, respectively. Likewise, excessively long irradiation times beyond optimal point can also result in a reduction in the anthocyanin extraction from grape pomace [124,125], blueberry peel [121], blackcurrant bagasse [51], black rice bran [45], black carrot pomace [39], and corn husk [43].

In general, the excessive intensification of MW extraction process parameters resulted in reducing anthocyanin extraction yields from agri-food by-products due to their degradation. In the literature, it is well established that anthocyanins are thermolabile compounds [115,137]. Several studies have shown that anthocyanins’ degradation rate augments as the temperature increases [138,139,140]. Thus, it is recommended to use non-thermal MW processes to extract anthocyanins, that is, extraction temperatures below 60 °C, to minimize losses due to thermal degradation. In addition to extraction temperature, extraction time also significantly impacts MW extraction. Overall, increased extraction times lead to a progressive release of target compounds from the sample matrix into the extraction solvent [19]. Nevertheless, longer extraction times do not always favor anthocyanin recovery. On the contrary, it can even cause their reduction. After a certain extraction time, the analyte concentrations in the sample and solvent reach a final equilibrium [19]. The excess time after this equilibrium can cause anthocyanin degradation due to higher exposure of these extracted compounds to severe conditions of the extraction procedure, such as high temperatures and microwave powers. However, anthocyanin degradation can occur even when non-thermal MW processes are employed and the irradiation times are optimized, mainly if high microwave powers are applied. When the microwave power is excessively high, internal overheating can occur, leading to carbonization and other reactions such as isomerization and/or degradation of sample components [129]. Indeed, high temperatures can be reached when samples are subjected to MW treatment. For example, extraction mixtures of coffee cherry peel by-product, bilberry pomace and orange peel, grape pomace, and wine lees achieved up to 89, 90, 93.5, and 117 °C during MW extraction, respectively [50,124,131,133,141]. Zhao et al. [137] reported that malvidin-3-glucoside and malvidin-3,5-diglucoside exposed to thermal and MW treatments shared some degradation products, demonstrating that the thermal pathway has a key role in the degradation of MW-treated anthocyanins. Sun et al. [115] and Zhao et al. [137] have deduced the thermal degradation pathways of anthocyanins. According to the authors, anthocyanins’ thermal degradation pathway occurs through the successive loss of sugar moieties (for anthocyanins with two or more sugar moieties) and subsequent opening of the C-ring, forming a carbinol pseudo base. Next, chalcone is formed by hydrolysis of the remaining sugar moiety and later cleaved between C2 and C3. Then, intermediate products from A-ring and B-ring are oxidized into different compounds depending on the anthocyanin. Other parallel thermal degradation pathways of anthocyanins are also described [115,137]. The thermal degradation pathway seems to be the main phenomenon related to anthocyanin degradation in MW-treated samples. However, anthocyanin degradation during MW extraction can be at least partially explained by oxidative phenomena. When extreme MW processing conditions are applied, decomposition reactions of water molecules can occur, leading to the increasing production of reactive oxygen species, especially hydroxyl radicals [137]. Indeed, it has been reported that the hydroxyl radicals formation is boosted as the microwave power and irradiation time increases [142]. Quan et al. [142] postulated that the oxidative degradation pathway of anthocyanins under MW treatment is given by the Baeyer–Villiger-type oxidation (for more details regarding this pathway, see Section 3.1). Therefore, the action of high temperatures together with reactive oxygen species on anthocyanins could explain their degradation during MW extraction, particularly when high microwave powers are employed.

In addition to MW process parameters, anthocyanins’ structural features also significantly impact their recovery from plant matrices. Herrman et al. [143] noticed that 3-deoxyanthocyanins (anthocyanin analogs that are unsubstituted at C3 of the C-ring) are more stable to MW treatment than common anthocyanins. The authors also pointed out that acylated anthocyanins were more stable than non-acylated ones [143]. Similar behavior was reported by Liazid et al. [144] in grape skin anthocyanins, in which MW treatment remarkably enhanced the acylated glucoside anthocyanin recovery regarding monoglucoside anthocyanins compared to the solid-liquid maceration classical method. Wang et al. [145] observed that delphinidin was more stable than petunidin under MW treatment. Meanwhile, Sivamaruthi et al. [146] verified that petunidin was more stable than cyanidin after MW exposure.

Finally, the employment of high-intensity MW technology to extract anthocyanins from agri-food by-products can lead to drastic anthocyanin degradation. However, in general, non-thermal MW treatments associated with low/moderate microwave powers result in higher anthocyanin yields without inducing any anthocyanin degradation/modification. Therefore, non-thermal MW treatment is a promising innovative technology to recover anthocyanins from agri-food by-products.

### 3.3. Ultrasound

The use of acoustic energy for recovering phytochemical compounds from plant matrices has strongly increased in the past decade due to its versatility in developing non-thermal extraction processes [147,148]. In this way, several thermolabile bioactive compounds such as phenolic compounds may have their biological activity maintained after this high-energy extraction technique [149]. Extracts obtained from ultrasound-based extraction processes have a high potential for developing nutraceuticals and functional food products. Furthermore, extraction processes based on ultrasound (US) technology enable low-cost industrial manufacturing lines, mainly due to the reduction in solvent used and extraction time, contributing to the lower energy expenditure [150]. US technology also meets one of the most critical demands from modern consumers to develop sustainable green processes [151,152]. Therefore, the combination of US-based extraction techniques using agri-food by-products is a promising approach for obtaining extracts rich in phenolic compounds such as anthocyanins.

US technology is based on the acoustic cavitation phenomenon observed in a liquid medium subjected to sonication treatment. The application of a low-frequency (16 to 100 kHz) and high-power (>10 W) ultrasound into liquid systems promotes the generation of acoustic fields which are associated with the occurrence of mechanical waves and vibrations [150,153]. The formation and subsequent collapse of microbubbles characterize the acoustic cavitation phenomenon. The acoustic cavitation microbubbles convert acoustic energy provided by the acoustic field into mechanical and thermal energies. The implosive collapse of microbubbles produces punctual zones of very high temperature (approximately 10,000 K) and pressure (about a few tens of GPa) [154]. Thus, during their collapse, shock waves and microjets are observed in the sonicated liquid system, resulting in turbulence and temperature rise. The acoustic cavitation shear stress effect on the surface of cells and particles is known as sonoporation [155,156]. Acoustic cavitation enables the formation of micropores and even the disruption of cellular structures. Therefore, the sonoporation effect is a powerful mechanism for extracting bioactive compounds from plant matrices because it favors the release of phytochemicals found inside plant cells into the extraction solvent [157]. Likewise, the sonication treatment also favors phytochemicals diffusion into the liquid medium due to the temperature increase and turbulence provided by acoustic cavitation [20,152]. However, US processing can generate free radicals such as hydroxyl radicals, promoting the degradation of bioactive compounds extracted from plant matrices [158]. The greater formation of these radicals is associated with the US process intensification. In this way, US-based extraction processes must be designed considering the opposite effects of acoustic energy on the recovering and degrading of target compounds concerning the generation of free radicals. On the other hand, the number of free anthocyanins can be reduced after US processing due to the formation of protein/carbohydrate-polyphenol conjugates induced by acoustic energy [159,160,161,162]. Xue et al. [163] induced the formation of conjugates between soy protein isolate and cyanidin-3-galactoside using an ultrasound intensity of 105.86 W/cm² for 20 min. However, the antioxidant capacity of proteins can be enhanced due to the formation of covalent conjugates with polyphenols [160].

Table 4 summarizes some interesting recent studies regarding the application of US technology to recover anthocyanins from agri-food by-products. Acoustic energy-based extraction processes are promising extraction techniques for phenolic compounds. Compared to conventional exhaustive extraction techniques based on solid-liquid extraction principles such as percolation, Soxhlet, and others. US-assisted extraction processes are more energy efficient regarding the shorter processing time. Another critical issue is related to green solvents, making these processes more sustainable and greener [164]. Grillo et al. [121] tested different natural deep eutectic solvents for the US-assisted recovery of anthocyanins from blueberry peels, the main by-product of blueberry fruit industrial processing. The authors emphasized the importance of developing new, environmentally friendly bio-based solvents to enhance the efficiency and selectivity of innovative extraction processes.

US technology presents anthocyanin degradation mechanisms similar to PEF and MW technologies (see Section 3.1 and Section 3.2, respectively). Chemical and thermal degradations are observed for anthocyanin molecules after applying acoustic cavitation treatment similarly to PEF and MW mechanisms, respectively. Tiwari et al. [165] reported that anthocyanin degradation during US processing occurs due to extreme mechanical and thermal stress generated by the acoustic cavitation phenomenon. The main US process variables are ultrasonic power, temperature, and processing time, which are directly associated with chemical and thermal degradations. The increase in ultrasonic power increases the acoustic cavitation intensity, contributing to the temperature rise and favoring the formation of free radicals. The use of an external source of heat to maintain a working temperature may also contribute to thermal degradation. In this sense, the development of non-thermal US-assisted extraction processes may preserve anthocyanins’ chemical stability. Processing time is associated with the exposition time of the plant material to mechanical and thermal stress due to the application of acoustic energy. Thus, longer processing times may be responsible for severe degradations in anthocyanins. Xue et al. [166] examined the one-way effect of the ultrasonic power (100 to 500 W), temperature (40 to 60 °C), and processing time (10 to 50 min) on the anthocyanin recovery from the raspberry by-product. For each US process variable, the authors observed an optimum condition of anthocyanin extraction yield (300 W, 50 °C, and 40 min) followed by an accentuated decrease, which certainly indicated the anthocyanin degradation. Otherwise, the US process intensification employing longer processing time did not promote anthocyanin degradation during their extraction from jabuticaba by-products at 50 W/L and 25 kHz after 40 min [167]. Thus, the US process intensification by increasing extraction parameters does not always lead to enhancements in anthocyanin extraction yields. Therefore, in this section, we focus on explaining the different US process conditions and their impact on energy delivered to the extraction system, since distinct results are observed for this innovative technology depending on process configuration.

In general, US treatment can be performed using an ultrasonic bath and ultrasonic probe. The main difference between them is associated with the source of acoustic energy. However, both configurations are based on a piezoelectric transducer as a generator of ultrasound power [168]. Ultrasonic probe-based systems are more employed to extract bioactive compounds than ultrasonic baths due to the higher ultrasonic intensity [150]. Higher ultrasound energy densities or specific energies are obtained from the ultrasonic probe because ultrasonic bath systems present a greater liquid volume medium, which reduces the ultrasonic energy performance for the same processing time. Thus, in ultrasonic probe-based systems, a more efficient cavitation effect is observed because the energy delivered is concentrated in a lower liquid volume [168]. Tarone et al. [53] studied the impact of ultrasound intensity (1.1, 3.7, 7.3, and 13.0 W/cm^2^) and solvent composition concerning water/ethanol ratio (0, 25, 50, 75, and 100 g water/100 g) on anthocyanin recovery from jabuticaba by-products using an ultrasonic probe of 13 mm at 19 kHz. Non-thermal US-assisted extractions were performed for 3 min with a maximum temperature of 33 °C. Ultrasound intensity had a positive effect on the extraction yield. In general, the ultrasound intensity increase promoted a greater mass transfer into the liquid medium. However, the solvent composition modulated the anthocyanin extraction from jabuticaba peel. The mixture of 50 g water/100 g of solvent was more efficient (up to 350%) compared to the extremes of solvent composition (0 and 100 g water/100 g). The US process intensification by increasing the acoustic cavitation intensity has a slight impact on anthocyanin recovery compared to solvent composition. These results suggest chemical degradation due to high mechanical and thermal stress combined with the greater formation of free radicals. Galván D’Alessandro et al. [54] examined the impact of US processing on anthocyanin degradation extracted from black chokeberry wastes employing a US water bath at 30.8 kHz. According to the results reported by them, anthocyanins were degraded after 60 min at 70 °C and ultrasound nominal power of 100 W using as solvent a mixture of 50% ethanol in water. The authors attributed to thermal effects the decrease in anthocyanin yield with processing time. Although Galván D’Alessandro et al. [54] had used an external source of heat to maintain their extraction conditions at 70 °C and observed a reduction in anthocyanin yield, they used an ultrasound water bath of 1 L. In contrast, Tarone et al. [53] used a probe of 13 mm inside a Falcon tube with 25 mL of solvent. The mode of application of the acoustic energy has a fundamental role in the ultrasound energy performance for the extraction. The acoustic energy amount delivered by the ultrasonic probe was applied into a small liquid volume compared to the ultrasonic bath, which presented a scale 40 times larger.

US-based extraction techniques have been combined with the use of enzymes to potentialize the rupture of the wall cell of plant materials. The modulation of the plant cell membrane permeability through sonoporation, which can promote even the cell lysis, is the most important effect associated with US treatment for the recovery of phytochemical compounds [166]. Zhang et al. [55] evaluated the US-assisted enzymatic extraction of anthocyanins from mulberry wine by-products using pectinase and pectinase compound enzymes. They optimized the technological conditions to increase anthocyanin recovery for functional food purposes. Optimum conditions for extraction were observed at 52 ℃, 315 W, 94 min, and using an enzyme dosage of 0.22%. The authors concluded that US-assisted enzymatic extraction is an efficient, economical, and environmentally friendly extraction technique. Likewise, Xue et al. [166] verified that the US-assisted enzymatic extraction technique was efficient and environmentally friendly for recovering cyanidin-3-glucoside with a purity of 93.46%, and cyanidin-3-rutinoside with a purity of 94.16% from raspberry wine by-products after 30 min at 44 °C and 290 W using a pectinase dosage of 0.16%. US-assisted enzymatic extraction technique was up to 177% more efficient than conventional hot water extraction technique regarding anthocyanin yield results.

Finally, from the recent studies concerning the application of US technology for recovering anthocyanins from agri-food by-products, we verified that this innovative technology presents a high potential to produce extracts with a high content of anthocyanins and other phenolic compounds. However, the mode of application of acoustic energy may lead to severe anthocyanin degradation mainly due to the intense mechanical and thermal stress associated with the acoustic cavitation phenomenon. The development of non-thermal US-assisted extractions is a promising alternative for reducing anthocyanin degradation.

## 4. Trends, Challenges, and Perspectives for Anthocyanins Application in Food Systems

Anthocyanins’ properties have allowed food scientists to apply them in food systems such as natural colorants, antioxidants, and smart packaging agents. Certainly, science and industries have evaluated the potential use of these biomolecules in different food systems and with different purposes. In this sense, we present a brief discussion about the traditional claims of the use of anthocyanins and the recent innovative uses of these phenolic compounds, as well as the current challenges for their implementation in food systems.

Anthocyanin water-soluble pigments are relatively unstable, with the greatest stability occurring under acidic conditions. Temperature, pH, and oxygen concentration are the variables that mainly affect their stability [174]. Food technologists have been developing new processes and applications to overcome these limitations. Fortunately, the pH of foods is on average between 4.0 and 6.5, while carbonated beverages and citrus and grape juices show pH ranging from 2.8 to 3.5. Furthermore, non-acylated anthocyanins can condense with themselves (self-association) or with other organic molecules (copigmentation), which tend to form more stable complexes (this occurs during winemaking, for example) [175].

Radishes, red potatoes, red cabbage, black carrots, purple corn, and purple sweet potatoes contain acylated anthocyanins with high stability during processing and storage when compared to non-acylated counterparts. These acylated molecules can be used as natural colorant agents, especially in juices and water-based systems with pH lower than 3 [174,176]. Furthermore, acylated anthocyanins showed high resistance to in vitro simulated gastrointestinal digestion compared to less complex anthocyanins from red wine [177,178]. This improves the bioavailability of anthocyanins and, as a consequence, their beneficial health effects. It demonstrates that despite the low stability of certain anthocyanins, we can use anthocyanins’ molecular structure features and their interaction with the medium and other organic macromolecules to overcome the stability issues [179,180,181].

An interesting example of color anthocyanins-based additives is the grape skin extract (European Economic Community, number E163), which contains mainly malvidin-3-glucoside. This color agent, commercially known as enocyanin, is restricted to color non-carbonated and carbonated drinks, beverage bases, and alcoholic beverages. Enocyanin (purplish-red liquid) is obtained from an aqueous extract of red grape pomace, a by-product, that remains after the grapes press process during juice manufacture [174]. Grape skin extract and its microencapsulated form have been applied as an antioxidant, antimicrobial, and health-promoting agent [182,183], demonstrating the multi-properties of anthocyanins. Eminol^®^ is another anthocyanin-rich extract obtained from red grape pomace through a patented extraction process (Grupo Matarromera, ES 2 309 032). This extract was added to four different food matrices: milkshake, custard dessert, omelet, and pancake. The authors observed that these matrices protect the anthocyanins against intestinal degradation, improving their bioaccessibility [184].

Black sorghum grain contains 3-deoxyanthocyanins that show higher heat resistance and stability over a wide pH range than C3-substituted anthocyanin analogs because of their particular structure. These biomolecules accumulate especially in the bran (a by-product of sorghum milling) of the black sorghum grain [143]. The luteolinidin, apigeninidin, and tricitinidin are examples of 3-deoxyanthocyanins. These compounds change color when exposed to electromagnetic radiation (photochromic properties), which has interested the food and pharmaceutical industries [185].

**Figure 3:**
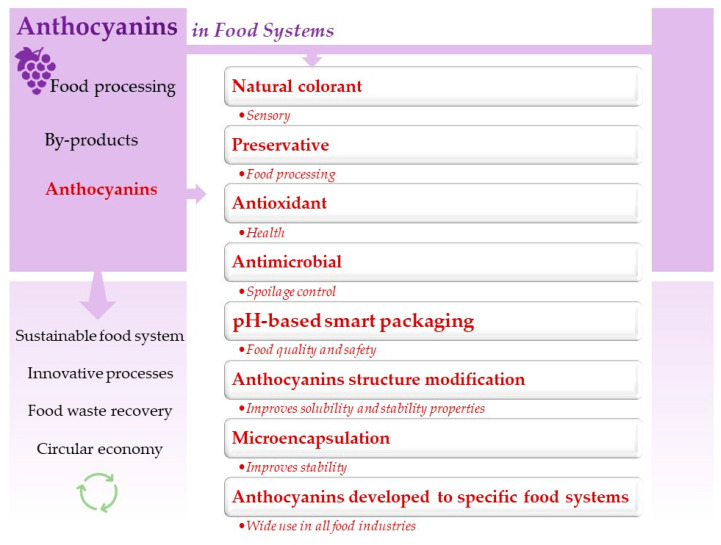
Anthocyanins can be recovered from food agri-food by-products. It is an important strategy to improve the food chain, especially to develop a sustainable food system, by using innovative processes and a food waste recovery approach. The second part of this figure (right) brings the anthocyanin uses commonly described by literature and industry, such as natural colorants, preservatives, antioxidants, antimicrobials, and health-promoting agents, as well as the innovative uses recently described in scientific reports; for instance, the pH anthocyanin-based smart packaging, and anthocyanin structure modification. Microencapsulation with macromolecules, such as proteins and polysaccharides, has been reported to enhance the anthocyanins’ stability during food processing and storage and over the human gastrointestinal digestion system. Food systems present different behavior and, as a consequence, the food technologists should carefully develop anthocyanin formulations appropriate to these specific systems using, for example, the strategies presented herein. Certainly, novel purposes will appear over time about these multi-property biomolecules. Nevertheless, the literature data clearly demonstrate that the food industry may extensively explore anthocyanins as food additives and functional ingredients.

Anthocyanins and their derivatives have been traditionally applied/reported in food systems to improve their sensory and stability properties during processing and storage. These biomolecules have been applied as natural colorants, preservatives, antioxidants, antimicrobials, and health-promoting agents, as well as in active and smart packaging and pH indicator systems (for more details, consult the reviews by Yong and Liu [186] and Echegaray et al. [17]). Figure 3 shows innovative uses, recently published in reports, of anthocyanins in food systems.

The dairy industry is constantly developing products, so it is not a surprise that the use of anthocyanins in dairy products has been evaluated in recent decades, especially as colorants and antioxidants agents. Furthermore, the biological activities of anthocyanins can be easily explored in the dairy industry by adding these phenolic compounds. Milk (α-, β- and κ-casein) and whey proteins (β-lactoglobulin e α-lactalbumin) can interact with anthocyanins by hydrophilic and hydrophobic groups, which have been associated with the increase in anthocyanin stability (thermal, light, and oxidative) during processing and storage. The protective effect of milk proteins on anthocyanin stability can be improved by preheating the milk. Preheated milk presented β-casein and β-lactoglobulin conformational structures that are more open, exposing the interior residues, enhancing the interaction between these proteins and anthocyanins [179,180,181].

Heated milk is recommended for yogurt processing because of the whey protein complex (formed in thermal treatment) depositing on casein micelles, which increases the process yield and product quality. In this way, the use of anthocyanins as a natural colorant in yogurts is interesting. The milk protein-anthocyanins complex and the pH of fermented milk (approximately 3.0–4.5) can increase the biomolecules’ stability, as previously mentioned [187]. Certainly, the temperature of preheating treatment affects the proteins’ structural conformations and, consequently, the binding affinity with the anthocyanins [188].

It is important to point out that food matrices can effectively protect anthocyanins during food processing/storage and over gastrointestinal digestion. Anthocyanins are stable during gastric digestion, but they show low intestinal stability because of the neutral pH (7.0). Anthocyanins are not essential nutrients, but their daily intake is an essential component of a healthy lifestyle that can confer protection against non-communicable diseases. Therefore, the bioavailability of anthocyanins has been evaluated in the past few years. The food components, such as proteins and polysaccharides, represent an interesting method to control the delivery of anthocyanins in the digestive tract and protect and anchor them in different types of use [184]. These chemical interactions between matrices’ components and anthocyanins can result in some variation in the hue of anthocyanins at different pHs and, as a consequence, affect the color variation, which is important, for example, in pH-indicator systems or milk-based beverages [189].

The bioavailability of anthocyanins is greatly reduced over gastrointestinal digestion, as previously mentioned. This directly affects the bioactivities of these biomolecules. Microencapsulation of anthocyanins can be an interesting strategy to improve their stability and delivery in the human body. Microparticles can be designed with different wall materials, such as casein, whey proteins, and polysaccharides exploring physicochemical properties of these macromolecules in digestive systems [190]. Furthermore, the intake of anthocyanins together with dietary lipids, such as coconut oil, can result in a protective effect of lipids on bioaccessible polyphenols. It seems that the stability and recovery of anthocyanins increase with fat content in the food matrix. This effect has been attributed to the hydrophobic interactions between anthocyanins and lipids. The anthocyanins incorporate into the lipid phase of the micelles, which increase their stability [191].

Recently, a research group from Australia presented an interesting way to use anthocyanins from red cabbage, building an active use by-date indicator for milk. They developed an anthocyanin-agarose film capable of changing its color in the presence of lactic acid from microbial metabolism. This allows the consumer to determine the quality of milk easily (fresh, spoiling, and spoiled milk) by the naked eye. The anthocyanin-agarose film indicates the quality of milk according to pH changes: fresh milk (blue color at pH 6.8–6.0), spoiling (purple color at pH 6.0–4.5), and spoiled milk (pink color at pH 4.0–4.5). The milk quality can be monitored in real time using this type of smart packaging, and this is very important to reduce food waste and improve the food supply chain [192]. Anthocyanins have been explored as freshness monitoring agents in, for instance, milk, fish, and pork meat [189]. Unlike milk, spoiled fish and pork meat show an increase in pH because of the release of volatile nitrogenous compounds, such as ammonia, during the spoilage process [11,193].

In addition to interaction with organic macromolecules, copigmentation, and shelf-association, anthocyanins’ structure can be chemically modified to improve their physicochemical properties, such as stability and solubility. Marathe et al. [194] enzymatically esterified purified anthocyanins from floral waste with lauric acid using lipase. This esterification improved the stability (thermal and oxidative) of anthocyanins compared to their non-esterified form. Furthermore, the anthocyanin fatty acid esters bio-synthetized showed enhanced lipophilicity, which allowed the authors to apply them in emulsion-based systems (e.g., cupcakes, sandwich biscuit cream, etc.). This approach can be interesting to be applied in dairy products, for example.

In this review, we reported the use of innovative processes (mild/non-thermal and environmentally friendly processes) to recover anthocyanins from agri-food by-products. Grape skin extract and 3-deoxyanthocyanins from the bran of black sorghum grain are excellent examples of obtaining anthocyanins from agri-food by-products. In this way, we can use cheap and abundant material to produce high-value goods and decrease the environmental impact promoted by food industry activities. Certainly, anthocyanin recovery from agri-food by-products is the first step of the challenge of using these biomolecules in food systems. The stability (in different food systems and over gastrointestinal digestion) and regulatory aspects are the main issues that should be the focus of scientists and the industry [23,195].

One challenging issue is to develop a food system in which the anthocyanins can be used as a natural colorant and, at the same time, the interaction between anthocyanins and food components does not alter their hue and protects them during processing/storage and over gastrointestinal digestion. For example, suppose anthocyanins are applied in a milk-based beverage. In that case, their color/hue may change, which can negatively affect the acceptability, but on the other hand, the interaction with milk proteins can improve their stability. The use of anthocyanins to simultaneously meet these purposes, ingredient/additive (natural colorant) and bioactive compound (biological properties), should be jointly approached. They have been evaluated separately, but we have enough technology to integrate both properties in only one food system.

The use of anthocyanins as additives brings new insights to the food industry because of their multiple properties. They can be used to meet technological criteria and simultaneously increase food’s functionalities. This is the most important advantage related to the use of natural compounds as food additives or active compounds in the food industry. In the era in which food technology and health sciences are walking together each day, the use of natural compounds to improve food properties is an inevitable trend. We have been reading in scientific articles that synthetic food additives should be replaced by natural ones because they are unsafe. Nonetheless, synthetic and natural food additives are both safe, according to regulatory aspects. Therefore, what drives this trend is the various technological and functional properties of natural compounds as compared with synthetic ones.

## 5. Conclusions

In this review, we showed that several human clinical trials have demonstrated the key role of anthocyanin intake as health-promoting agents by preventing different non-communicable chronic diseases (e.g., diabetes, obesity, dyslipidemias, metabolic syndrome, cancers, neurodegenerative disorders, and cardiovascular diseases, among others) and promoting overall wellbeing. The beneficial health effects of anthocyanins are related to their capacity of modulating gut microbiota composition/activity and signaling pathways involved in glucose and lipid metabolism, inflammation, oxidative damage, autophagy, and apoptosis. In addition to being employed as bioactive/drug agents, market trends have pointed out the use of anthocyanins in food systems as natural colorants, antioxidants, preservatives, antimicrobials, and active and smart packaging components (e.g., pH-sensing films). The great deal of evidence showing the beneficial health effects coupled with the growing market demand for natural additives has boosted the search for new anthocyanin sources. In this context, different agri-food by-products, particularly berry wastes generated from the fruit juicing process, proved to be cheap, feasible, and available sources for obtaining anthocyanins-rich extracts. Innovative mild/non-thermal processing technologies (e.g., PEF, MW, and US) have been demonstrated to be effective, rapid, low-cost, and eco-friendly methods to recover anthocyanins from agri-food by-products. However, it should be emphasized that the process conditions’ intensity together with parameters combination and mode of application can significantly compromise the anthocyanin extraction yields due to the oxidative and/or thermal degradation that occur when in overly intensive conditions. Another issue that deserves attention in future studies is the need for the development/enhancement of technologies that increase anthocyanins’ stability during food processing/storage and over gastrointestinal digestion to improve their applicability, bioavailability, and bioactivity.

## Figures and Tables

**Table 1:** A summary of human trials showing the potential health beneficial effects of anthocyanins.

Anthocyanin	Anthocyanin Dose	Time	Subject Profile	Study Type	Major Findings	Reference
MEDOX^®^ (Purified anthocyanins) ^a^	320 mg/day	12 weeks	160 subjects with prediabetes or early untreated T2DM (40–75 years; 54 males and 106 females)	Randomized, double-blind, placebo-controlled trial	- ↓ HbA1c, LDL-c, and ApoB; - ↑ ApoA1; - ↓ insulin resistance; - More effective at improving glycemic control, insulin sensitivity, and lipids profile among patients with elevated metabolic markers.	[68]
MEDOX^®^ (Purified anthocyanins) ^a^	320 mg/day	12 weeks	138 subjects with prediabetes or newly diagnosed T2DM (40–75 years; 45 males and 93 females)	Randomized, double-blind, placebo-controlled trial	- ↑ adipsin and ApoA1; - ↓ visfatin, HbA1c, C-peptide, C-peptide index, and ApoB.	[69]
MEDOX^®^ (Purified anthocyanins) ^a^	320 mg/day	12 weeks	121 patients with fasting hyperglycemia (average age: 61 years old; 42 males and 79 females)	Randomized controlled trial	- ↑ IGFBP-4 fragments; - ↓ FGB and postprandial C-peptide; - ↓ LDL-c and ApoB.	[70]
MEDOX^®^ (Purified anthocyanins) ^a^	320 mg/day	4 weeks	14 healthy (35.2 ± 3.16 years old; 8 males and 6 females), 14 T2DM at-risk (50.1 ± 3.15 years old; 8 males and 6 females), and 12 T2DM (57.7 ± 2.5 years old; 8 males and 4 females) individuals	Open-label design	- ↓ FGB, LDL-c, and uric acid in the T2DM at-risk group; - ↓ IL-6, IL-18, and TNF-α in the T2DM group.	[71]
MEDOX^®^ (Purified anthocyanins) ^a^	20, 40, 80, 160, and 320 mg/day	14 days	111 healthy adults (18–35 years old; 39 males and 72 females)	Randomized, double-blind, placebo-controlled trial	- ↓ FBG; - ↓ IL-6, IL-10, and 8-iso-PGF_2α_; - IL-10 and 8-iso-PGF_2α_ decreased with increasing anthocyanin dose.	[72]
MEDOX^®^ (Purified anthocyanins) ^a^	40, 80, and 320 mg/day	12 weeks	169 dyslipidemic subjects (35–70 years old; 45 males and 124 females)	Randomized, double-blind, placebo-controlled trial	- ↑ SOD activity in the high dose group after 6 weeks (320 mg/day); - IL-6, TNF-α, 8-iso-PGF_2α_, 8-OHdG, and MDA decreased with increasing anthocyanin dose.	[65]
MEDOX^®^ (Purified anthocyanins) ^a^	40, 80, and 320 mg/day	12 weeks	176 dyslipidemic subjects (35–70 years old; 46 males and 130 females)	Randomized, double-blind, placebo-controlled trial	- ↓ plasma levels of 6 ceramide species (Cer 16:0, 18:0, 20:0, 22:0, 24:0 and 24:1) in a dose-dependent manner; - Cer 16:0 and Cer 24:0 reduction was correlated with the decreases in non-HDL-c, ApoB and TC in the high dose group (320 mg/day).	[73]
MEDOX^®^ (Purified anthocyanins) ^a^	40, 80, and 320 mg/day	12 weeks	176 dyslipidemic subjects (57.41 ± 7.95 years old; 46 males and 130 females)	Placebo-controlled, double-blind, randomized trial with multiple doses	- ↑ cholesterol efflux capacity, HDL-c, and ApoA1 in the high dose group (320 mg/day); - Cholesterol efflux capacity, HDL-c, and ApoA1 increased with increasing anthocyanin dose.	[74]
MEDOX^®^ (Purified anthocyanins) ^a^	320 mg/day	24 weeks	150 hypercholesterolemic subjects (40–65 years old; 63 males and 87 females)	Randomized, double-blind, placebo-controlled trial	- ↓ NAP-2, ENA-78, IL-8, SDF-1α, and MCP-1; - ↓ LDL-c; - ↑ HDL-c; - ↓ hs-CRP, IL-1β, and soluble P-selectin.	[75]
MEDOX^®^ (Purified anthocyanins) ^a^	320 mg/day	4 weeks	12 lean (33.0 ± 3.2 years old; 6 males and 6 females), 9 overweight (49.9 ± 4.2 years old; 5 males and 4 females), and 8 obese (43.3 ± 4.5 years old; 4 males and 4 females) participants	Clinical trial	- ↓ MCP-1 across all groups; - ↓ IL-6 in the obese group; - Trend for reducing TNF-α across all groups.	[76]
MEDOX^®^ (Purified anthocyanins) ^a^	320 mg/day	4 weeks	51 subjects (25 normal subjects (38.2 ± 2.7 years old; 13 males and 12 females) and 26 MetS subjects (56.6 ± 2.6 years old; 14 males and 12 females))	Clinical trial	- ↓ FBG, TG, and LDL-c in the MetS group; - ↓ hs-CRP in the MetS group; - ↓ ADP-induced platelet activation (↓ P-selectin expression) in the MetS group.	[77]
MEDOX^®^ (Purified anthocyanins) ^a^	320 mg/day	4 weeks	35 subjects (15 normal subjects (37.3 ± 2.9 years old; 10 males and 5 females) and 20 MetS subjects (56.2 ± 2.9 years old; 11 males and 9 females))	Clinical trial	- ↓ FBG, TC, TG, and LDL-c in the MetS group; - ↑ PPAR-γ expression in the MetS group; - ↓ hs-CRP, TNF-α, IL-6, and IL-1A in the MetS group; - ↓ COX-2 and PECAM-1 in both groups; - ↑ SOD in the MetS group.	[78]
MEDOX^®^ (Purified anthocyanins) ^a^	320 mg/day	4 weeks	26 pro-thrombotic overweight and obese individuals (39 ± 11 years old; 9 males and 17 females)	Randomized, double-blind, placebo-controlled, crossover design dietary intervention trial	- ↓ ADP-induced platelet activation-related conformational change and degranulation (↓ PAC-1 and P-selectin expression); - ↓ thrombogenic progression (↓ monocyte-platelet aggregate formation and PECAM-1 expression); - ↓ platelet aggregation, collagen, and arachidonic acid.	[79]
MEDOX^®^ (Purified anthocyanins) ^a^	320 mg/day	4 weeks	16 sedentary pro-thrombotic individuals (38 ± 12 years old; 3 males and 13 females)	Randomized, double-blind, placebo-controlled, cross-over design dietary intervention trial	- ↓ ADP-induced platelet activation-related conformational change and degranulation (↓ PAC-1 and P-selectin expression); - ↓ thrombogenic progression (↓ monocyte-platelet aggregate formation and PECAM-1 expression).	[80]
MEDOX^®^ (Purified anthocyanins) ^a^	320 mg/day	16 weeks	27 individuals with MCI (*n* = 8) or stable non-obstructive coronary artery disease (*n* = 19) (55–70 years old; 18 males and 9 females)	Open-label study	- ↓ RANTES; - Improved verbal memory function (learning, recall, and recognition) and cognitive speed.	[81]
Anthocyanins-rich blackcurrant extract	150, 300, and 600 mg	Acute	14 men and 9 postmenopausal women (46 ± 14 years old) consuming a high-carbohydrate meal	Randomized, double-blind, crossover trial	- ↓ postprandial glycemia, serum insulin, and serum GIP in the high dose group (600 mg).	[82]
Anthocyanins-rich blackcurrant extract	3.2 mg/kg/day (~240 mg/day)	5 weeks	34 healthy individuals (38 ± 11 years old; 21 males and 13 females)	Double-blind placebo-controlled trial design	- ↓ post-exercise-induced MDA and plasma ROS-generating capability; - ↓ post-exercise plasma TNF-α and IL-6; - ↑ post-exercise plasma IL-10; - ↑ salivary mucosal anti-bacterial defense proteins (BD2 and secretory IgA).	[66]
Anthocyanins-rich black soybean testa extract	31.45 mg/day	8 weeks	63 overweight or obese individuals (30.59 ± 9.25 years old; 50 males and 30 females)	Randomized, double-blinded, placebo-controlled clinical trial	- ↓ waist circumference, hip circumference, TG, LDL-c, and non-HDL-c; - ↓ TC/HDL-c and LDL-c/HDL-c.	[83]
Anthocyanins-rich Queen Garnet plum juice	200.8 mg/day	4 days	16 overweight subjects (65.9 ± 6.0 years old; 3 males and 13 females) consuming a high-fat high energy meal	Crossover, randomized, controlled, double-blind clinical trial	- ↑ 2 h postprandial flow-mediated dilatation and microvascular post-occlusive reactive hyperemia; - ↓ hs-CRP and IL-6.	[84]
Anthocyanins-rich Queen Garnet plum juice	47 and 201 mg/day	8 weeks	31 subjects with MCI (75.3 ± 6.9 years old; 12 males and 19 females)	Randomized, controlled, double-blind clinical trial	- ↓ TNF-α in the high dose group (201 mg/day).	[85]
Anthocyanins from blueberry	258 mg/day	16 weeks	37 older adults with MCI (≥68 years old; 17 males and 20 females)	Randomized, double-blind, parallel groups, placebo-controlled trial	- Improved semantic access and visual-spatial memory; - Trend for enhanced psychomotor speed of processing; - Cognitive benefits were correlated with parent anthocyanin compounds.	[86]
Anthocyanins-rich black rice extract	19.08 mg/day	12 weeks	48 subjects with subjective memory impairment (63.88 ± 7.59 years old; 16 males and 32 females)	Double-blind randomized, placebo-controlled trial	- Improved subjective memory; - Trend for improving cognitive function.	[87]
Anthocyanins-rich cherry juice	138 mg/day	12 weeks	49 older adults with mild-to-moderate dementia (≥70 years old; 25 males and 24 females)	Randomized controlled clinical trial	- Improved verbal fluency, short-term memory, and long-term memory; - ↓ Systolic blood pressure.	[88]
Anthocyanins-rich bilberry extract	840 mg/day	6 weeks	13 patients with current mild-to-moderate ulcerative colitis (19–61 years old; 10 males and 3 females)	Open, prospective, non-blinded, and non-controlled pilot trial	- ↓ IFN-γ-receptor 2, IFN-γ, and TNF-α expression in the colon; - ↓ phosphorylated (activated) p65-NF-κB in the colon; - ↓ STAT1 expressing cells; - ↓ serum MCP-1 and TNF-α; - ↓ Th17-specific cytokine protein expression (IL-22) in the colon; - ↑ IL-10 expression in the colon.	[67]
Anthocyanins-rich blackcurrant extract	210 mg/day	7 days	14 older adults (73.3 ± 1.7 years old; 6 males and 8 females)	Randomized, double-blind, placebo-controlled, crossover design study	- ↓ carotid-femoral pulse-wave velocity, central blood pressure, brachial systolic blood pressure, brachial mean blood pressure, brachial pulse pressure, and augmentation index.	[89]
Anthocyanins-rich red fruit juice ^b^	205.5 mg/day	8 weeks	57 healthy male volunteers (20–50 years old)	Prospective, randomized, placebo-controlled parallel design	- ↑ NQO-1 and HO-1 transcript levels in peripheral blood lymphocytes; - Modified microbial community; - ↑ relative abundance of beneficial bacteria *Adlercreutzia*; - Potentially harmful taxa were not enriched.	[90]
Anthocyanins-rich aronia extract	30 mg/day	12 weeks	66 healthy male volunteers (18–45 years old)	3-arm, randomized, double-blind, placebo-controlled, parallel trial	- ↑ flow-mediated dilation; - Modulated gut microbiota composition; - ↑ growth of butyrate-producing bacteria *Anaerostipes*.	[91]
Anthocyanins-rich bilberry extract	88.5 mg/day	12 weeks	109 healthy adults (20–60 years old; 34 males and 75 females)	Randomized, double-blind, placebo-controlled, parallel-group comparison trial	- ↓ post-visual display terminal load HFC-1; - Ameliorated and relieved the tonic accommodation of ciliary muscles caused by visual display terminal tasks and near-vision tasks.	[92]
Delphinol^®^ (Purified anthocyanins) ^c^	60 mg/day	8 weeks	16 healthy female subjects (27–57 years old)	Randomized, double-blind, placebo-controlled pilot study	- ↑ skin brightness and collagen content; - ↓ facial skin redness.	[93]

Where: ↓ indicates reduction; ↑ indicates increase; *8-iso-PGF_2α_*, 8-iso-prostaglandin F_2α_; *8-OHdG*, 8-hydroxy-2′-deoxyguanosine; *ADP*, adenosine diphosphate; *Apo*, apolipoproteins; *BD2*, beta-defensin 2; *COX-2*, cyclooxygenase 2; *ENA-78*, epithelial neutrophil-activating peptide; *FBG*, fasting blood glucose; *GIP*, glucose-dependent insulinotropic polypeptide; *HbA1c*, glycated hemoglobin A1c; *HDL-c*, high-density lipoprotein cholesterol; *HFC-1*, high-frequency component 1; *HO-1*, heme oxygenase 1; *hs-CRP*, high-sensitivity C-reactive protein; *IFN-γ*, interferon-gamma; *IgA*, immunoglobulin A; *IGFBP-4*, insulin-like growth factor binding protein 4; *IL*, interleukins; *LDL-c*, low-density lipoprotein cholesterol; *MCI*, mild cognitive impairment; *MCP-1*, monocyte chemoattractant protein 1; *MDA*, malonaldehyde; *MetS*, metabolic syndrome; *NAP-2*, neutrophil-activating peptide 2; *NF-κB*, nuclear factor-kappa B; *NQO-1*, NAD(P)H quinone oxidoreductase 1; *PAC-1*, procaspase 1; *PECAM-1*, platelet endothelial cell adhesion molecule 1; *PPAR-γ*, peroxisome proliferator-activated receptor gamma; *RANTES*, regulated on activation, normal T-cell expressed and secreted; *ROS*, reactive oxygen species; *SDF-1α*, stromal cell-derived factor 1-alpha; *SOD*, superoxide dismutase; *STAT1*, signal transducer and activator of transcription 1; *T2DM*, type 2 diabetes mellitus; *TC*, total cholesterol; *TG*, triglycerides; *TNF-α*, tumor necrosis factor-alpha. ^a^ The MEDOX^®^ food supplement capsules (Medapalett Pharmaceuticals, Biolink, Sandnes, Norway) contain purified anthocyanins isolated from bilberries (*Vaccinium myrtillus*) and blackcurrant (*Ribes nigrum*) (33.0% of 3-*Ο*-β-glucosides, 3-*Ο*-β-galactosides, and 3-*Ο*-β-arabinosides of cyanidin; 58.0% of 3-*Ο*-β-glucosides, 3-*Ο*-β-galactosides, and 3-*Ο*-β-arabinosides of delphinidin; 2.5% of 3-*Ο*-β-glucosides, 3-*Ο*-β-galactosides, and 3-*Ο*-β-arabinosides of petunidin; 2.5% of 3-*Ο*-β-glucosides, 3-*Ο*-β-galactosides, and 3-*Ο*-β-arabinosides of peonidin; 3.0% of 3-*Ο*-β-glucosides, 3-*Ο*-β-galactosides, and 3-*Ο*-β-arabinosides of malvidin; and 1.0% of 3-*Ο*-rutinoside of cyanidin and delphinidin). ^b^ The anthocyanins-rich red fruit juice (Eckes-Granini GmbH, Niederolm, Germany) was produced from red grape juice, lingonberry juice from concentrate, apple, blueberry, and strawberry puree, Aronia juice from concentrate, and acerola puree (100% fruit content). The total anthocyanin content of red fruit juice was 274 mg/L, comprising 33% of malvidin-3-glucoside, 14.3% of cyanidin-3-galactoside, 11.6% of peonidin-3-glucoside, 10.3% of petunidin-3-glucoside, 7.7% of delphinidin-3-glucoside, 6.8% of cyanidin-3-arabinoside, 6.4% of cyanidin-3-glucoside, 3.8% of delphinidin-3-arabinoside, 2.5% of malvidin-3-galactoside, 2% of petunidin-3-galactoside, and 1.6% of delphinidin3-galactoside. ^c^ The Delphinol^®^ food supplement capsules (Oryza Oil & Fat Chemical Co., Ltd., Aichi, Japan) contain purified anthocyanins isolated from maqui berry (*Aristotelia chilensis*). Each capsule contains ≥ 35% anthocyanin glycosides and ≥25% delphinidin glycosides.

**Table 2:** Summary of the main recent findings showing the effects of the pulsed electrical field (PEF) technology on anthocyanin extraction from agri-food by-products.

Waste/By-Product	Extraction Process Parameters	Major Findings	Reference
Raspberry by-product	*Electric field intensity:* 1 and 3 kV/cm *Specific energy input:* 1, 6, and 12 kJ/kg *Frequency:* 20 Hz *Pulse width:* 20 μs	- Improved anthocyanin extraction (up to 25.7%); - PEF process intensification did not increase anthocyanin extraction; - Mild PEF (1 kV/cm and 6 kJ/kg) was sufficient to achieve higher anthocyanin extraction.	[47]
Sweet cherry by-product	*Electric field intensity:* 0.5, 1, and 3 kV/cm *Specific energy input:* 10 kJ/kg *Frequency:* 5 Hz *Pulse width:* 20 μs	- No effect on the number and type of anthocyanins extracted; - Improved anthocyanin extraction (up to 38.4%); - PEF process intensification did not increase anthocyanin extraction; - Cyanidin-3-glucoside content reduced as the electric field intensity increased.	[48]
Sour cherry by-product	*Electric field intensity:* 1, 3, and 5 kV/cm *Specific energy input:* 10 kJ/kg *Frequency:* 10 Hz *Pulse width:* 20 μs	- Improved anthocyanin extraction (up to 54%); - PEF process intensification did not increase anthocyanin extraction.	[99]
Blueberry by-product	*Electric field intensity:* 10–35 kV/cm *Pulse number:* 2–14 *Pulse width:* 2 μs	- PEF process intensification improved anthocyanin extraction (up to 20 kV/cm and 10 pulses); - High electric field intensity (>20 kV/cm) and pulse number (>10 pulses) drastically reduced anthocyanin extraction; - PEF technology was more effective than US.	[100]
Blueberry by-product	*Electric field intensity:* 3 kV/cm *Specific energy input:* 1, 5, and 10 kJ/kg *Frequency:* 10 Hz *Pulse width:* 20 μs	- No effect on the number and type of anthocyanins extracted; - Improved anthocyanins extraction (up to 75%); - Anthocyanin extraction increased with PEF process intensification; - No evidence of individual anthocyanin degradation due to PEF application.	[46]
Blueberry by-product	*Electric field intensity:* 1, 3, and 5 kV/cm *Specific energy input:* 10 kJ/kg *Frequency:* 10 Hz *Pulse width:* 20 μs	- Improved anthocyanin extraction (up to 111%); - Anthocyanin extraction increased with PEF process intensification.	[101]
Blueberry by-product	*Electric field intensity:* 1, 3, and 5 kV/cm *Specific energy input:* 10 kJ/kg *Frequency:* 10 Hz *Pulse width:* 20 μs	- No effect on the number and type of anthocyanins extracted; - Improved anthocyanin extraction (up to 95%); - Anthocyanin extraction increased with PEF process intensification.	[102]
Blueberry pomace	*Electric field intensity:* 10, 15, and 20 kV/cm *Specific energy input:* up to 41.03 kJ/kg *Pulse number:* 10, 50, and 100 *Pulse width:* 2 μs	- Anthocyanin extraction increased with PEF process intensification; - PEF technology was more effective than US and high voltage electrical discharges.	[103]
Peach pomace	*Electric field intensity:* 0.8–10 kV/cm *Specific energy input:* 0.02–20 kJ/kg *Frequency:* 0.1 Hz *Pulse width:* 4 μs	- Improved anthocyanin extraction (up to 11.8-fold); - PEF process intensification significantly reduced anthocyanin extraction.	[98]
Grape pomace	*Electric field intensity:* 1.2, 1.8, and 3.0 kV/cm *Specific energy input:* 18 kJ/kg *Pulse number:* 200–2000 *Pulse width:* 100 μs	- Improved anthocyanin extraction (up to 18.9%); - The increase in electric field intensity (1.2–3.0 kV/cm) had no effect on anthocyanin extraction.	[104]
Grape pomace	*Electric field intensity:* 13.3 kV/cm *Specific energy input:* 0–564 kJ/kg *Frequency:* 0.5 Hz	- Improved anthocyanin extraction (up to 5.3-fold); - Anthocyanin extraction increased with PEF process intensification; - PEF technology was more effective for anthocyanin extraction than US (up to 22%) and high voltage electrical discharges (up to 55%).	[105]
Grape peel	*Specific energy input:* 289.8 (PEF-I) and 37.8 W (PEF-II) *Pulse number:* 25.2 (PEF-I) and 9.7 (PEF-II) *Frequency:* 10 Hz *Pulse width:* 6 μs	- Improved anthocyanins extraction (up to 4-fold); - PEF-I treatment was more effective for anthocyanin extraction than US.	[106]
Plum peel	*Specific energy input:* 228 (PEF-I) and 17.8 W (PEF-II) *Pulse number:* 25.2 (PEF-I) and 9.7 (PEF-II) *Frequency:* 10 Hz *Pulse width:* 6 μs	- PEF technology was not able to increase anthocyanin extraction compared to control.	[106]

**Table 3:** Summary of the main recent findings showing the effects of the microwave (MW) technology on anthocyanin extraction from agri-food by-products.

Waste/By-Product	Extraction Process Parameters	Major Findings	Reference
Blueberry peel	*Microwave power:* 500 W *Temperature:* 40–100 °C *Irradiation time:* 2–40 min *Solvent:* Choline chloride:lactic acid (1:1) containing 25% (*v*/*v*) water	- Maximum anthocyanin extraction was achieved at 60 °C and 15 min; - Improved anthocyanin extraction (up to 62.7%); - MW can degrade anthocyanins during the extraction process.	[121]
Blueberry bagasse	*Microwave power:* 525 and 700 W *Irradiation time:* 3 min *Solvent:* Acidified water	- MW process intensification reduced anthocyanin extraction.	[122]
Fig peel	*Microwave power:* 400 W *Temperature:* 40–115 °C *Irradiation time:* 5–35 min *Solvent:* Acidified hydroethanolic mixtures (0–100% ethanol)	- Maximum anthocyanin extraction was achieved at 5 min, 64.21 °C, and 100% ethanol; - MW technology was more effective for anthocyanin extraction than thermal (38%) and US extraction (16.73%); - MW process intensification can reduce anthocyanin extraction.	[123]
Eggplant peel	*Microwave power:* 100–300 W *Irradiation time:* 5–15 min *Solvent:* Acidified hydroethanolic mixtures (55–95% ethanol)	- Anthocyanin extraction improved as the microwave power increased and irradiation time and ethanol concentration reduced; - Maximum anthocyanin extraction was achieved at 298.84 W, 5.78 min, and 55.56% ethanol; - MW process intensification can reduce anthocyanin extraction.	[40]
Black soybean seed coat	*Microwave power:* 340–680 W *Irradiation time:* 2.5–7.5 min *Solvent:* Hydroethanolic mixtures (20–60% ethanol)	- Maximum anthocyanin extraction was achieved at 510 W, 7.5 min, and 60% ethanol; - Improved anthocyanin extraction (up to 4.72-fold); - Microwave power intensification (>510 W) caused anthocyanin degradation.	[42]
Grape pomace	*Microwave power:* 600–1000 W *Irradiation time:* 5–10 min *Solvent:* Acidified water	- Overall, MW process intensification improved anthocyanin extraction; - Irradiation time intensification (>7 min) caused anthocyanin degradation.	[124]
Grape pomace	*Microwave power:* 300–600 W *Irradiation time:* 1–3 min *Solvent:* Water	- Maximum anthocyanin extraction was achieved at 428 W and 2.23 min; - MW process intensification can reduce anthocyanin extraction.	[125]
Grape pomace	*Microwave power:* 100–300 W *Irradiation time:* 10–15 min *Solvent:* Choline chloride:citric acid (2:1) containing 10–50% (*v*/*v*) water	- Anthocyanin extraction enhanced as the microwave power increased and irradiation time decreased; - Maximum anthocyanin extraction was achieved at 300 W, 10 min, and 30% water.	[126]
Bilberry pomace	*Microwave power:* 300–600 W *Irradiation time:* 1–16 min *Solvent:* Solvent-free	- Anthocyanin recovery enhanced as the microwave power increased; - The higher the microwave power, the shorter the irradiation time required for anthocyanin extraction; - Maximum anthocyanin extraction was achieved at 600 W and 6.5 min.	[50]
Sour cherry peel	*Microwave power:* 350–500 W *Irradiation time:* 0.5–1.5 min *Solvent:* Acidified hydroethanolic mixtures (20–80% ethanol)	- MW process intensification improved anthocyanin extraction; - Maximum anthocyanin extraction was achieved at 500 W, 1.5 min, and 80% ethanol.	[49]
Blackcurrant bagasse	*Microwave power:* 385–700 W *Irradiation time:* 10–20 min *Solvent:* Acidified hydroethanolic mixtures (0–90% ethanol)	- Maximum anthocyanin extraction was achieved at 551 W, 16.4 min, and 60% ethanol; - MW process intensification can reduce anthocyanin extraction.	[51]
Peach pomace	*Microwave power:* 180–900 W *Irradiation time:* 10–50 s *Solvent:* Hydroethanolic mixture (70% ethanol)	- Improved anthocyanin extraction (up to 26-fold); - Anthocyanin extraction enhanced as the microwave power reduced and irradiation time increased; - Maximum anthocyanin extraction was achieved at 180 W and 50 s.	[127]
Onion peel	*Microwave power:* 700–1000 W *Irradiation time:* 3–5 min *Solvent:* Hydroethanolic mixtures (40–75% ethanol)	- Maximum anthocyanin extraction was achieved at 700 W, 5 min, and 75% ethanol; - MW process intensification can reduce anthocyanin extraction.	[44]
Black rice bran	*Microwave power:* 298–800 W *Irradiation time:* 13–147 s *Solvent:* Acidified water	- Maximum anthocyanin extraction was achieved at 648 W and 83 s; - MW process intensification can reduce anthocyanin extraction.	[45]
Black carrot pomace	*Microwave power:* 340–680 W *Irradiation time:* 5–15 min *Solvent:* Hydroethanolic mixtures (10–30% ethanol)	- Maximum anthocyanin extraction was achieved at 348 W, 9.86 min, and 19.8% ethanol; - MW technology was more effective for anthocyanin extraction than conventional (133%) and US extraction (24.12%); - Microwave power intensification reduced anthocyanin extraction.	[39]

**Table 4:** Summary of the main recent findings showing the effects of the ultrasound (US) technology on anthocyanin extraction from agri-food by-products.

Waste/By-Product	Extraction Process Parameters	Major Findings	Reference
Blueberry peel	*Ultrasound nominal power:* 100 and 500 W *Processing time:* 40 min *Temperature:* 40 °C *Solvent:* Five natural deep eutectic solvents *Ultrasonic equipment:* Probe at 20 kHz	- The US-based extraction technique achieved 21.18 mg/g total anthocyanin content after 30 min of sonication at 500 W power while conventional extraction based on a stirring and heating system extracted 22.70 mg/g after 2 h at 55 °C and 200 rpm.	[121]
Blueberry pomace	*Ultrasound nominal power:* 400 W *Processing time:* 15–35 min *Temperature:* 50–70 °C *Solvent:* Acidified hydroethanolic mixture (70% ethanol) *Ultrasonic equipment:* Probe	- Delphinidin-3-*O*-glucoside, delphindin-3-*O*-arabinoside, petunidin-3-*O*-glucoside, cyanidin-3-*O*-arabinoside, cyanidin-3-*O*-glucoside, malvidin-3-*O*-glucoside, and malvidin-3-*O*-arabinoside were recovered; - Compared to conventional solvent extraction, US-assisted extraction resulted in higher anthocyanin recovery.	[169]
Jabuticaba by-product	*Ultrasound intensity:* 1.1–13.0 W/cm² *Processing time:* 3 min *Solvent:* Hydroethanolic mixtures (0–100% ethanol) *Ultrasonic equipment:* Probe at 19 kHz with a diameter of 13 mm	- The higher ultrasound intensities presented the lowest values for bioactive compounds and antioxidant capacity; - Extracts presented a brownish color indicating degradation of their compounds and loss of color due to the increase in the cavitation effect; - Solvent composition had a strong influence on anthocyanin recovery.	[53]
Jabuticaba by-product	*Power density:* 50 and 60 W/L *Processing time:* 10–40 min *Solvent:* Acidified water (pH 1.5, 3.0, and 7.0) *Ultrasonic equipment:* Ultrasound bath at 25 and 40 kHz	- Ellagic acid and cyanidin-3-*O*-glucoside were the major phenolic compounds extracted from jabuticaba by-product; - Processing time and solution pH were the most significant variables.	[167]
Black chokeberry waste	*Ultrasound nominal power:* 0–100 W *Processing time:* 0–240 min *Temperature:* 20–70 °C *Solvent:* Hydroethanolic mixtures (0–50% ethanol) *Ultrasonic equipment:* Ultrasound bath at 30.8 kHz	- At high temperatures, anthocyanin yield was decreased with processing time suggesting their thermal degradation.	[54]
Blackberry waste	*Ultrasound nominal power:* 1500 W *Processing time:* 15 min *Temperature:* 4 °C *Solvent:* Water *Ultrasonic equipment:* Probe at 20 kHz with a diameter of 25 mm	- Ultrasonicated blackberry waste had low antioxidant compounds compared to blackberry waste. However, these compounds showed a high in vitro bioaccessibility.	[170]
Blackberry by-product	*Ultrasound nominal power:* 580 W *Processing time:* 90 min *Temperature:* 80 °C *Solvent:* Hydroethanolic mixtures (50% and 70% ethanol) and acidified water (pH 2) *Ultrasonic equipment:* Ultrasound bath at 37 kHz	- Hydroethanolic mixtures were more efficient to extract anthocyanins; - Cyanidin-3-*O*-glucoside, cyanidin-3-*O*-rutinoside, cyanidin-3-*O*-malonyl-glucoside, and cyanidin-3-*O*-dioxalylglucoside were identified in the extracts.	[52]
Pomegranate by-product	*Ultrasound nominal power:* 70–210 W *Duty cycle:* 20–80% *Processing time:* 1–10 min *Solvent:* Hydroethanolic mixture (50% ethanol) *Ultrasonic equipment:* Probe at 20 kHz	- The pulsed US-assisted extraction of bioactive compounds from pomegranate peel was an emerging green, energy, and time-efficient extraction process for the extraction of food bioactives; - Multicriterial numerical optimization suggested 116 W sonication power with 80% duty cycle for 6 min for extraction of 22.51 mg cyanidin-3-glucosides/100 g pomegranate peel.	[171]
Eggplant by-product	*Ultrasound nominal power:* Not specified *Processing time:* 15–45 min *Temperature:* 25 and 50 °C *Solvent:* Acidified hydroethanolic mixtures (70% and 96% ethanol) *Ultrasonic equipment:* Ultrasound bath	- Five anthocyanins were identified: delphinidin-3-*O*-rutinoside-5-glucoside, delphinidin-3-*O*-glucoside, delphinidin-3-*O*-rutinoside, cyanidin-3-*O*-rutinoside, and petunidin-3-*O*-rutinoside; - US-assisted extraction was preferable to conventional solid-liquid extraction due to the lower temperature used and higher delphinidin 3-*O*-rutinoside content.	[41]
Eggplant by-product	*Ultrasound nominal power:* Not specified *Processing time:* 15–45 min *Temperature:* 50–70 °C *Solvent:* 50–90% (*v*/*v*) methanol or 2-propanol in water *Ultrasonic equipment:* Ultrasound bath at 12.5, 25, and 37.5 kHz	- Solvent concentration exhibited a negative effect on the anthocyanin content; - Ultrasonic frequency, processing time, and extraction temperature had a positive effect on the anthocyanin recovery.	[172]
Grape pomace	*Ultrasound nominal power:* 150–300 W *Processing time:* 2.5–10 min *Temperature:* 25–55 °C *Solvent:* Water *Ultrasonic equipment:* Probe at 20 kHz with a diameter of 13 mm	- The aqueous extracts of grape by-product presented higher levels of antioxidant capacity (1.4-fold), anthocyanins (1.3-fold), and total phenolic (1.2-fold) by comparing with the conventional extraction technique.	[173]
Raspberry by-product	*Ultrasound nominal power:* 100–500 W *Pectinase dosage:* 0.05–0.25% *Processing time:* 10–50 min *Temperature:* 40–60 °C *Solvent:* Acidified hydroethanolic mixture (60% ethanol) *Ultrasonic equipment:* Probe	- Cyanidin-3-glucoside with a purity of 93.46% and cyanidin-3-rutinoside with a purity of 94.16% were obtained from raspberry by-products; - The optimum extraction parameters were obtained at 44 °C, 290 W, 30 min, and pectinase dosage of 0.16%.	[166]
Mulberry by-product	*Ultrasound nominal power:* 200–400 W *Pectinase dosage:* 0.15–0.25% *Processing time:* 60–120 min *Temperature:* 40–60 °C *Solvent:* Acidified water *Ultrasonic equipment:* Ultrasound bath	- The optimum extraction conditions were 52 ℃, 315 W, 94 min, and enzyme dosage of 0.22%; - Cyanidin-3-*O*-glucoside and cyanidin-3-*O*-rutinoside are the two main anthocyanins in mulberry by-product.	[55]
Peach waste	*Ultrasound nominal power:* 80–400 W *Processing time:* 20–120 s *Temperature:* 25–55 °C *Solvent:* Hydroethanolic mixture (70% ethanol) *Ultrasonic equipment:* Probe at 24 kHz with a diameter of 10 mm	- Lower ultrasound powers and longer processing times contributed to a greater anthocyanin recovery.	[127]

## Data Availability

Not applicable.
